# Machine learning-based evaluation of seed priming and biostimulant applications in rainfed wheat

**DOI:** 10.7717/peerj.20578

**Published:** 2026-03-02

**Authors:** Leila Sharifi, Mahdi Ghiyasi, Bardia Talebian, Younes Rezaee Danesh, Solmaz Najafi, Murat Tunçtürk, Rüveyde Tunçtürk, Beatrice Farda, Loretta Giuseppina Pace

**Affiliations:** 1 Department of Computer Engineering, Faculty of Electric and Computer Engineering, Urmia University, Urmia, Iran; 2 Department of Plant Production and Genetics, Faculty of Agriculture, Urmia University, Urmia, Iran; 3 Department of Plant Protection, Faculty of Agriculture, Van Yuzuncu Yil University, Van, Turkey; 4 Department of Field Crops, Faculty of Agriculture, Van Yuzuncu Yil University, Van, Turkey; 5 Department of Life, Health and Environmental Sciences, University of L’Aquila, L’Aquila, Italy

**Keywords:** *Triticum aestivum*, Nanoparticles, Micronutrients, Linear regression, Ridge regression, Least absolute shrinkage and selection operator, ElasticNet regularization, Support vector regression, Random forest, Extreme gradient boosting

## Abstract

**Background:**

Rainfed wheat suffers from water scarcity and micronutrient deficits, calling for innovative practices. This study tests zinc sulphate (ZnSO_4_) seed priming combined with foliar iron (Fe), zinc (ZN), and manganese (Mn) (various formulations) and uses multiple machine-learning models to predict agronomic outcomes.

**Methods:**

A field experiment was conducted in northwestern Iran using a factorial randomized complete block design with four replications. Treatments included three ZnSO_4_ priming concentrations (0.1%, 0.2%, 0.3%) and five foliar sprays (conventional, magnetized, and nano-formulations of Fe, Zn, and Mn), plus water controls. Agronomic traits (*e.g.*, spike emergence, plant height, yield, protein content) were measured. Data were analyzed with ANOVA and modelled using eight regression algorithms (Linear, Ridge, Lasso, Elastic Net, support vector regression (SVR), Random Forest, eXtreme Gradient Boosting (XGBoost), CatBoost).

**Results:**

The results demonstrated that seed priming with ZnSO_4_ considerably expedited spike emergence, enhanced plant height, and increased biological yield, with elevated ZnSO_4_ concentrations intensifying these effects. Foliar application of biostimulants enhanced yield components, grain yield, and protein-related characteristics. The maximum grain production (1,648 kg ha^−1^) was attained with 0.3% ZnSO_4_ seed priming in conjunction with nano foliar application of Fe, Zn, and Mn, indicating synergistic nutrient uptake/use or, alternatively, the potential of prediction approaches such as regularized regressions (Ridge, Lasso, Elastic Net) were most accurate for biological yield, protein content, and harvest index, whereas XGBoost/CatBoost captured nonlinearities but were less consistent for seed-related features. Overall, ZnSO_4_ priming combined with nano biostimulants markedly enhances rainfed wheat performance.

## Introduction

Wheat (*Triticum aestivum* L.) is one of the most important strategic crops worldwide, playing a crucial role in ensuring global food security and it is primarily cultivated under rainfed conditions. However, this type of cultivation is facing multiple challenges, especially in arid and semi-arid regions (Gawdiya et al., 2024). Given the significance of this crop, adopting innovative approaches such as seed priming, foliar application of biostimulants, and advanced technologies like magnetized treatment and nanotechnology-based treatments offers effective solutions for enhancing wheat performance under rainfed conditions (Singha et al., 2024).

Seed priming, an advanced agricultural technology, is crucial in enhancing crop performance under rainfed conditions. This technique involves soaking seeds in specific nutrient solutions under controlled time and environmental conditions, which activates seed metabolism without initiating complete germination (Mawale, Nandini & Giridhar, 2024). Additionally, seed priming enhances the plant’s defence mechanisms against environmental stresses. Consequently, this adaptation enhances the plant’s resistance to drought and other environmental stresses (Ghiyasi et al., 2024). Furthermore, mineral-based seed priming, such as zinc sulphate, enables direct nutrient uptake by seeds, improving seedling growth in nutrient-deficient soils (Harris & Mottram, 2024).

Zinc (Zn) is an essential plant element, playing a role in over 300 enzymatic activities. It can enhance enzymes’ activity, such as superoxide dismutase, carbonic anhydrase, and RNA polymerase, which are involved in oxidative stress reduction, CO_2_ fixation, and gene expression regulation, respectively (Sánchez-Palacios et al., 2023).

Foliar application is another effective method for supplying essential nutrients in rainfed agriculture. Under rainfed conditions, where limited soil moisture restricts nutrient uptake by the roots, foliar application of micronutrients such as iron, zinc, and manganese can compensate for these deficiencies. Direct absorption of nutrients through the cuticle and stomata enables rapid translocation to photosynthetic tissues, enhancing chlorophyll production and improving the photosynthetic rate (Ghiyasi et al., 2023). Additionally, foliar application regulates stomatal opening and closure, optimising gas exchange and increasing drought resistance and grain yield under rainfed conditions. These benefits establish foliar application as an essential tool for nutritional management in rainfed farming.

The foliar application of nanoparticle-based biostimulants is notably recognised as a practical approach for improving the growth and yield of rainfed crops (Sodhi et al., 2025). Nanoparticles, thanks to their small size and large surface area, are absorbed more efficiently than conventional materials, penetrating leaf tissues quickly and releasing nutrients gradually for sustained uptake (Meena, Jat & Jain, 2021). For example, foliar application of zinc can boost the activity of photosynthesis-related enzymes, leading to increased biomass production and higher grain yield (Hao et al., 2021; Firozabad, Ghiyasi & Amirnia, 2024; Ghiyasi et al., 2019). Also, iron (Fe) is crucial in chlorophyll synthesis, electron transport, and plant respiration. Iron deficiency results in leaf chlorosis and reduced photosynthesis. Foliar application of iron, particularly in calcareous soils, enhances chlorophyll synthesis and improves plant growth (Saquee et al., 2023). Manganese (Mn) is involved in activating enzymes related to photosynthesis and redox reactions, and its deficiency can reduce sugar formation and weakens resistance to biotic and abiotic stresses. Its foliar application supports metabolic processes and enhances plant resilience (Zulfiqar et al., 2021). Magnetic water can modify solution physicochemistry (*e.g.*, lower surface tension, alter ionic mobility), thereby improving foliar penetration *via* cuticle and stomata. Reported effects include higher foliar absorption efficiency, enhanced photosynthesis, and stimulation of nutrient-metabolizing enzymes, with increased N–P–K uptake translating into better growth and yield under both optimal and stress conditions (Radhakrishnan, 2019; Putti et al., 2023). Additionally, magnetised water improves osmotic regulation by reducing surface tension, enhancing enzyme activity, and strengthening cell walls, while also boosting stress-response mechanisms, effects that are especially beneficial under rainfed water stress conditions (Zhao et al., 2022).

Although the physiological benefits of these treatments are well-documented, their collective evaluation under rainfed systems remains limited, particularly when combined with data-driven tools for performance forecasting. Linear regression models and their extensions (Ridge and Lasso regressions, and Elastic Net) are effective for predicting crop performance from climatic variables while reducing overfitting in complex datasets (Herrera et al., 2018; Setiya, Nain & Satpathi, 2023). For nonlinear relationships, advanced models such as Support Vector Regression (SVR), Random Forest, and eXtreme Gradient Boosting (XGBoost) are preferred due to their ability to handle high-dimensional, heterogeneous agricultural data, showing strong performance in yield prediction, plant health assessment, and environmental analysis. More complex models, including SVR, Random Forest (RF), and XGBoost (XGB), are extensively utilised for analysing nonlinear and intricate agricultural datasets. For example, SVR employs kernel functions to map nonlinear data into higher-dimensional feature spaces, proving valuable for predicting agricultural metrics such as water consumption and soil temperature (Vahidi, Shafian & Frame, 2025). RF and XGB leverage ensemble decision-making and gradient-boosting algorithms. In fact, they are favoured for their capacity to handle large, heterogeneous datasets and their robustness against outliers and noise (Yang et al., 2021; Parashar et al., 2024).

Although seed priming, foliar biostimulants, and machine learning have been extensively studied in agriculture, they have rarely been evaluated in combination. Most existing works either focus on agronomic practices to improve yield or on predictive modelling of crop traits, without linking the two. The joint assessment of physiological treatments under rainfed stress conditions together with machine learning-based prediction remains largely unexplored. This study integrates both aspects to achieve two main objectives. First, it assesses the direct agronomic and physiological effects of seed priming and foliar treatments on wheat. Second, it identifies the most effective predictive algorithms for analyzing complex trait interactions. Together, these findings provide a comprehensive framework for optimizing rainfed wheat management. This study hypothesised that seed priming and foliar biostimulants would enhance rainfed wheat performance and that machine learning models would predict these responses more accurately than traditional linear methods. Zinc sulphate priming at different concentrations and foliar application of iron, zinc, and manganese in conventional, nano, and magnetised forms were tested. Resulting data were modelled using multiple machine learning regressors to identify the most effective predictive approach.

## Materials & Methods

This study investigated the effects of seed priming with zinc sulphate and foliar application of iron, zinc, and manganese in conventional, subjected to a magnetic field, and nano-formulations on rainfed wheat (cv Azar 2) during the 2023 growing season. The experiment was conducted at the Urmia University Research Farm (37°10′N, 45°21′E, altitude 1,328 m) during the 2022–2023 growing season. The average daily temperature during the growing season was 14.8 ± 3.6 degrees Celsius, with a minimum and maximum average of 6.2 and 23.5 degrees. The average relative humidity was 58 ± 7% and the cumulative rainfall was 312 mm. Seed priming was performed at a temperature of 25 ± 2 degrees and a humidity of 60 ± 5%. Foliar spraying was also performed in the cool morning hours (8–10 AM) and at a temperature of 17–22 degrees and a humidity of 55–65% to minimize rapid evaporation of the solution and crystallization of salts. These temperature and humidity conditions are consistent with the climate of the rainfed regions of northwestern Iran and similar research on wheat.

### Experimental design

The experimental design followed a factorial arrangement within a randomised complete block design with two factors and four replications. The first factor involved seed priming with zinc sulphate (ZnSO_4_) at three concentrations (0.1%, 0.2%, and 0.3%), with not primed seeds as the control. Factor 2 comprised five foliar application treatments: conventional, subjected to a magnetic field, and nano-formulations of iron, zinc, and manganese at 2% concentrations, alongside magnetized treatment and conventional water as controls. Conventional and magnetized treatment utilized ZnSO_4_, FeSO_4_, and MnSO_4_, while nano-formulations employed zinc oxide nanoparticles (ZnO; average particle size 25 nm, specific surface area 50 m^2^ g^−1^), iron oxide nanoparticles (Fe_2_O_3_; 30 nm, 60 m^2^ g^−1^), and manganese oxide nanoparticles (MnO; 45 nm, 40 m^2^ g^−1^). Seed priming with concentrations of 0.1, 0.2 and 0.3% zinc sulfate and foliar application of micronutrients iron, zinc and manganese at two parts per thousand (0.2%) were selected based on physiological and biochemical grounds. Concentrations of zinc less than 0.1% do not have sufficient stimulating effect on antioxidant enzyme activity (SOD, CAT, APX) and auxin synthesis, while concentrations higher than 0.3% may cause osmotic stress and damage to the seed coat. The range of 0.1–0.3% has been reported as the optimal physiological range for stimulating seed metabolism and increasing germination uniformity. Also, the concentration of two parts per thousand was selected for foliar application due to the balance between absorption efficiency, physicochemical stability and physiological safety of the leaf; This level increases absorption through the cuticle and stomata, improves the activity of the enzymes nitrate reductase and carbonic anhydrase, and increases the concentration of Zn and Fe in the leaf without causing toxicity or leaf necrosis. Magnetized water for foliar applications was prepared using a laboratory-grade water magnetizer (Fanavary Iranian Pazhuhesh Nasir Company, FAPAN, Iran). The device employs a fixed, permanent magnet to generate a magnetic field of 0.5 T, magnetizing the water stream. Magnetization was conducted at 24 °C, with a production capacity of ∼20 L min^−^^1^. Unless otherwise specified, all foliar treatments labeled as ‘magnetic water’ were prepared under these conditions; control (non-magnetized) water was sourced identically but bypassed the magnetization unit. Each plot covered 12 m^2^, and foliar applications were administered at three growth stages: stem elongation, pre-anthesis, and anthesis (early grain filling). Applications were timed during cooler morning hours (before 10 AM) to minimise evaporation and optimise absorption. Weather forecasts were monitored to avoid post-application rainfall and leaching. The field had previously been cultivated with rainfed chickpeas. Pre-experiment soil analysis (0–30 cm depth) revealed an electrical conductivity (EC) of 0.45 dS m^−1^, pH 7.8, organic carbon content of 0.74%, and available nitrogen (0.06%), phosphorus (393.8 ppm), and potassium (21.08 ppm). Sowing occurred in early November using a seed drill at a density of 300 seeds/m^2^ (equivalent to 120 kg ha^−1^). Seeds were treated with 2% Tebuconazole (Baitan) before planting. Planting depth was set at four cm, and the first effective rainfall post-sowing marked the official sowing date. Fertilisation followed local recommendations: 180 kg ha^−1^ urea, 120 kg ha^−1^ triple superphosphate, and 90 kg ha^−1^ potassium sulphate. Phosphorus and potassium fertilisers were applied pre-sowing, half the nitrogen was applied at sowing, and the remainder was applied during stem elongation, timed with rainfall. Broadleaf and grass weeds were controlled using Topik (1 L ha^−1^) and Granstar (20 g ha^−1^) herbicides during tillering. Harvesting was performed manually.

### Crop monitoring and growth parameters evaluated

#### Spike emergence

Spike emergence was recorded as the number of days from sowing to the appearance of spikes from the flag leaf sheath. Observations were made daily, and the date on which 50% of the plants in a plot showed spike emergence was recorded (Liu et al., 2022).

#### Plant height

Plant height was measured at physiological maturity from the base of the plant at soil level to the tip of the spike, excluding awns. Measurements were taken from ten randomly selected plants per plot, and the average value was used for analysis (Zhang et al., 2023).

#### Spike density per square meter

Spike density was determined by counting the number of spikes within a 1 m^2^ area in each plot at the peak of heading. Counts were performed in multiple randomly selected locations per plot, and the mean value was calculated (Wen et al., 2022).

#### Number of seeds per spike

To assess the number of seeds per spike, mature spikes were randomly collected from each plot. The total number of seeds per spike was counted manually, and the average was recorded (Chu et al., 2023).

#### Thousand-Seed Weight (TSW)

A random sample of 1.000 fully matured, cleaned, and dried seeds was weighed using a precision balance. The measurement was expressed in grams (Milivojević et al., 2022).

#### Seed yield

Seed yield was determined by harvesting plants from a pre-measured area within each plot. The harvested grain was cleaned, dried to a standard moisture content of 12–14%, and weighed. The final yield was extrapolated to a per-hectare basis (kg ha^−1^) (Anbes & Asredie, 2025).

#### Biological yield

The biological yield was estimated by harvesting the total above-ground biomass from a known area within each plot. The plant material was dried to a constant weight before weighing to determine the total biomass yield (Lamlom, Irshad & Mosa, 2023).

#### Protein percentage and yield

Grain protein content was determined using the Kjeldahl method after milling. Protein yield was calculated by multiplying the grain protein percentage by the seed yield (Yan et al., 2022).

#### Harvest Index (HI)

The Harvest Index was calculated as the ratio of economic yield (grain yield) to the total biological yield. It was expressed as a percentage using the following formula (Broberg et al., 2021):

\begin{eqnarray*}HI= \frac{\mathrm{''}Grain~Yield\mathrm{''}}{\mathrm{''}Total~above-Ground~Biomass} \ast 100. \end{eqnarray*}

### Data modelling

Eight regression algorithms were employed for data modeling, Including CatBoost (CB), Elastic Net Regularization (EN), Least Absolute Shrinkage and Selection Operator (Lasso), Linear Regression (LR), Random Forest (RF), Ridge Regression (Ridge), Support Vector Regression (SVR), and Extreme Gradient Boosting (XGB) (Table S2). These algorithms were deliberately selected to provide a comprehensive evaluation of predictive performance, balancing model simplicity, interpretability, and capacity to capture complex non-linear relationships. Linear models (Ridge, Lasso, ElasticNet, LR) were included as baseline approaches for modeling linear relationships with controlled regularization, while SVR introduced flexibility through kernel functions to address potential non-linear dependencies. Random Forest represented tree-based ensemble methods that efficiently handle feature selection and interaction effects, whereas boosting algorithms (XGB and CB) were incorporated as state-of-the-art methods known for superior performance on structured agronomic datasets. Data were randomly partitioned into training and testing sets, with 50% allocated to machine learning model training and 50% reserved for model validation and accuracy assessment. While we used a 50% training/50% testing split, this choice is appropriate for relatively small datasets to ensure that both training and testing sets have sufficient data points. The main requirement is that the model has enough data to learn effectively, and the test set is large enough to reliably assess performance. Importantly, we also applied 5-fold cross-validation for all models. This procedure evaluates model performance across multiple splits, mitigates overfitting, and provides a more robust assessment than a single train–test split. To evaluate the model’s accuracy, two indicators, root mean square error percentage (RMSPE) and coefficient of determination (R^2^), were used. Analyses were conducted in Python using the following packages (versions for reproducibility): scikit-learn 1.2.2, XGBoost 2.0.3, CatBoost 1.2.7, NumPy 1.26.4, and Pandas 2.2.3. The percentage of root mean square error in the evaluation of various regression models is an important indicator in evaluating the efficiency and accuracy of the model. Unlike RMSE, which is scale-dependent, RMSPE emphasizes relative deviations, providing a clearer interpretation of proportional prediction accuracy. This indicator discusses the model’s error rate in predicting actual values. This criterion is formulated in the following equation. Where xi is the actual value and xl is the predicted value, and N is the number of observations.

\begin{eqnarray*}\mathrm{RMSPE}=100\sqrt{ \frac{\sum ({x}_{i}-{x}_{l})^{2}}{N} }. \end{eqnarray*}

The coefficient of determination (R^2^), which practically indicates the proportion of the variance of the actual data explained by the model, is also an important indicator for evaluating regression models. The values of this indicator are between zero and one, and the closer its value is to one, the more variance is explained by the model and the higher its accuracy. This criterion is formulated in the following equation. RSS represents the sum of squares of the residuals, and TSS represents the sum of squares.

\begin{eqnarray*}{R}^{2}=1- \frac{RSS}{TSS} . \end{eqnarray*}

To ensure transparency, reproducibility, and model robustness, a comprehensive preprocessing and optimization pipeline was applied to all input features prior to model training. Quantitative variables with missing values were imputed using column means, while categorical variables were encoded using ordinal encoding. Outliers in numeric features were clipped using the 1.5  × IQR method to reduce the influence of extreme values. Features with very low correlation (Spearman correlation <0.05) were excluded to reduce redundancy, and correlation heatmaps were generated for visualization. For linear models (LR, Ridge, Lasso, ElasticNet) and SVR, features were standardized using StandardScaler (zero mean, unit variance), whereas tree-based models (Random Forest, XGBoost, CatBoost) were applied directly to unscaled features.

To further enhance model performance and reduce overfitting, hyperparameter tuning was applied to all models using RandomizedSearchCV combined with five-fold cross-validation. This approach ensured that each algorithm was optimized for generalization rather than memorization of the training data, with particularly notable improvements observed for XGBoost and CatBoost due to their sensitivity to parameter settings. Together, these preprocessing and optimization procedures ensured that the reported results were based on models that were both accurate and reliable. SAS version 9.4 (SAS Institute Inc., Cary, NC, USA) software was used to statistically analyse the experiment’s data. Initially, the data were checked for normality using the Shapiro–Wilk test. Then, analysis of variance (ANOVA) was performed based on a randomised complete block design as a factorial design to determine the significance of the treatments’ effects on the studied traits.

The statistical model used in variance analysis was as follows:

\begin{eqnarray*}{Y}_{ij}=\mu +{T}_{i}+{e}_{ij} \end{eqnarray*}

Yij:

The observation of the i-th treatment in the j-th replication.

µ: The overall mean of the population.

Ti: The fixed effect of the i-th treatment.

eij: The random error term associated with the i-th treatment in the j-th replication.

After confirming the significance of the treatment effects at the 1% error level, the means were compared using the Fisher least significant difference (LSD) test.

### SHapley Additive exPlanations (SHAP) analysis

To determine which predictors most strongly influenced the prediction of each of the 11 traits in this study, feature importance analysis was performed using SHapley Additive exPlanations (SHAP) values. For linear models (Lasso, Ridge, and EN), the LinearExplainer was applied, whereas tree-based models (RF and CB) were interpreted using the TreeExplainer. For XGB, due to the incompatibility with TreeExplainer, the general SHAP Explainer was used instead. Finally, for SVR, the KernelExplainer was implemented on a random sample of 100 test instances to reduce computational cost. SHAP analyses were conducted across all traits using all the regression models introduced in this study.

## Results

Table 1 shows the results of the variance analysis of research data. Statistical analysis showed that the effects of seed priming and foliar spraying on all studied traits were significant at the 1% probability level. In addition, the interaction between the two factors was also significant, except for the number of spikes per square meter.

**Table 1:** Results of the analysis of variance (ANOVA) on experimental data.

	***Mean Square***										
***SV***	***df***	***SE***	***DM***	***PH***	***SM***	***SP***	***TW***	***SY***	***BY***	***PP***	***PY***	***HI***
R	3	5.16	1.4	0.1	36.6	1.8	0.2	133.2	12,200	0.4	1.2	0.7
A	3	237.8^**^	498.9^**^	52.6^**^	1,831.8^**^	316.5^**^	31.7^**^	16,384^**^	85,162^**^	0.7^**^	692.6^**^	4.3^**^
B	4	290.8^**^	314.0^**^	77.6^**^	4,623.1^**^	933.7^**^	34.9^**^	163,614^**^	283,983^**^	17.0^**^	11,036.6^**^	25.7^**^
A×B	12	3.6^**^	3.9^**^	1.8^**^	21.5 ^ns^	5.8^*^	0.5^**^	367^**^	47,689^**^	0.1^**^	25.4^**^	2.2^**^
Error	57	58.3	1.2	0.1	13.6	3.0	0.06	96	13,486	0.3	9.9	0.7
CV%		0.7	0.6	0.4	1.3	3.6	0.6	0.7	2.7	1.0	1.9	2.4

**Notes.**

SV Source of Variation df Degree of freedom SE Spike emergence (day) DM Day to Harvesting PH Plant height SM Number of Spikes (m^2^) SP Number Seeds per spike TW Thousand seed weight (g) SY Seed yield (Kg ha^−1^) BY Biological Yield (Kg ha^−1^) PP Protein (%), Protein yield (Kg ha^−1^) HI Harvest index R Replication A Seed priming B Foliar application A × B interaction of A and B CV% Coefficient of Variation ns non significant

* *p* < 0.05.

** *p* < 0.01.

### Spike emergence

**Figure 1:**
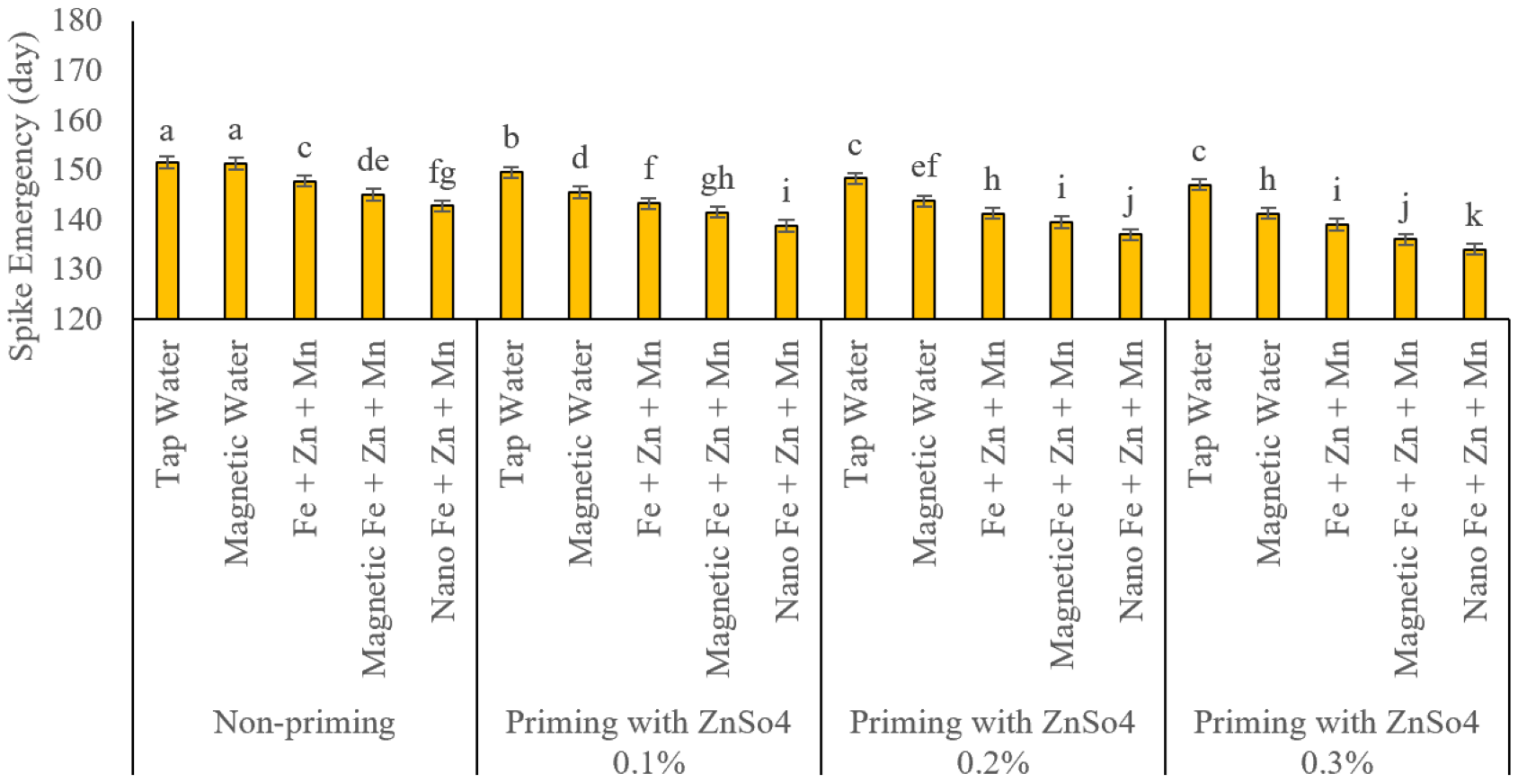
Comparison of the average interaction effects of seed priming with zinc sulphate at different concentrations and foliar spraying of iron, zinc, and manganese (at 2‰), in conventional, magnetic, and nano formulations on spike emergence (days). Means were separated by Least Significant Differences (LSD) at *p* = 0.05; groups sharing at least one letter are not significantly different (*p* > 0.05).

Figure 1 reports data on Spike emergence. Analysing the interactive effects of seed priming and foliar application A, the comparative analysis of the interactive effects of seed priming and foliar application demonstrated that seed priming with zinc sulphate (ZnSO_4_) significantly accelerated spike emergence. The data indicated that increasing the concentration of zinc sulphate led to a statistically significant reduction in the time required for spike emergence. The most extended duration for spike emergence was observed in treatments with seed priming and foliar application using regular and magnetised water, recorded at 151.5 and 151.25 days, respectively. Under seed priming conditions, foliar application of micronutrients significantly accelerated spike emergence. However, the type of treatment played a crucial role in this effect. Among the treatments, foliar application of nano-formulated iron, zinc, and manganese at a 2% concentration exhibited the most pronounced reduction in spike emergence time. Without seed priming, this treatment resulted in spike emergence occurring 142.75 days after sowing. Following this, foliar application of magnetised nutrient solutions reduced the emergence time to 145 days after sowing compared to control treatments, thereby shortening the transition to the reproductive phase. Conventional foliar application of iron, zinc, and manganese also significantly reduced spike emergence time (147.75 days after sowing) compared to the untreated control in the absence of seed priming. However, its effect was statistically less pronounced than the nano-formulated and magnetised nutrient solutions. A similar pattern was observed in treatments involving seed priming with zinc sulphate (ZnSO_4_). However, under seed priming conditions, across all concentrations, foliar application of magnetised water significantly shortened the duration required for the transition to the reproductive phase and spike emergence in the studied rainfed wheat variety compared to regular water application. Furthermore, increasing the concentration of zinc sulphate further reduced spike emergence time statistically significantly. Nonetheless, across all seed priming concentrations, the shortest spike emergence times were achieved through, in descending order, nano-formulated, magnetised, and conventional foliar applications of iron, zinc, and manganese. Among all studied treatments, the shortest spike emergence duration was recorded in the seed priming treatment with 0.3% zinc sulphate, where spikes emerged 134 days after sowing—a statistically significant reduction compared to all other treatment combinations.

### Day to harvesting

**Figure 2:**
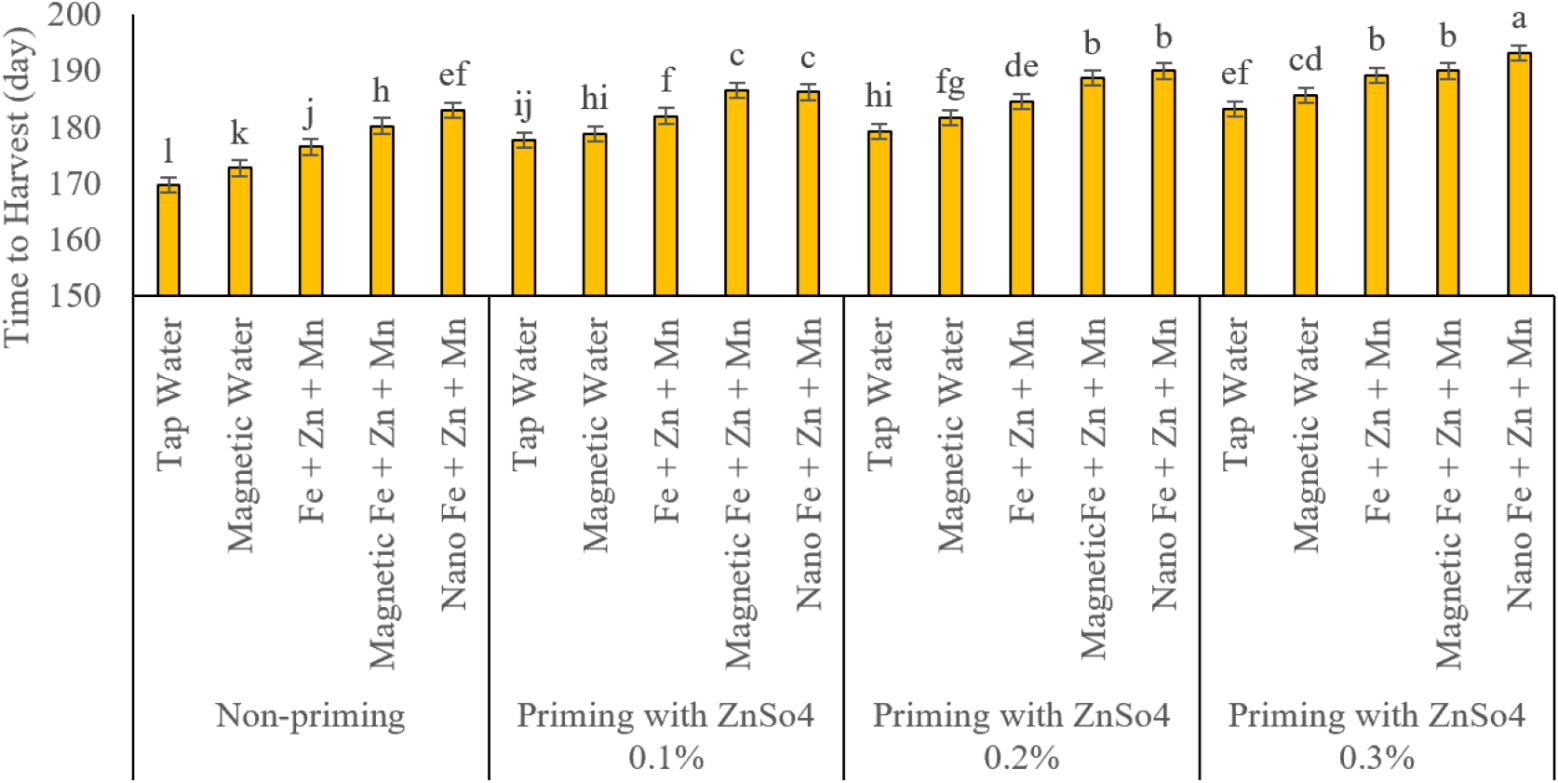
Comparison of the average interaction effects of seed priming with zinc sulphate at different concentrations and foliar spraying of iron, zinc, and manganese (at 2‰), in conventional, magnetic, and nano formulations time to harvest (day). Means were separated by Least Significant Differences (LSD) at *p* = 0.05; groups sharing at least one letter are not significantly different (*p* > 0.05).

Figure 2 depicts the results of the time needed to harvest the crop. The application of priming with zinc sulphate (ZnSO_4_) and foliar spraying with iron, zinc and manganese nutrients in all forms studied and foliar spraying with magnetized treatment water significantly increased the time from planting to harvest. The results also indicate that the required harvest time increases considerably with increasing zinc sulphate concentration. In addition, at none of the concentration levels of seed priming, foliar spraying treatments with iron, zinc, and manganese did not have statistically similar results, and foliar spraying treatments based on nanomaterials had significantly longer harvest time compared to other foliar spraying methods. Also, based on the data recorded from this experiment, foliar spraying with magnetized treatment water compared to conventional water led to an increase in the duration of crop harvest. The highest harvest time of 195.25 days was obtained from seed priming treatment with zinc sulphate (ZnSO_4_) at a concentration of 0.3% and foliar spraying of iron, zinc, and manganese in nano form, which was significantly higher than other treatments.

### Plant height

**Figure 3:**
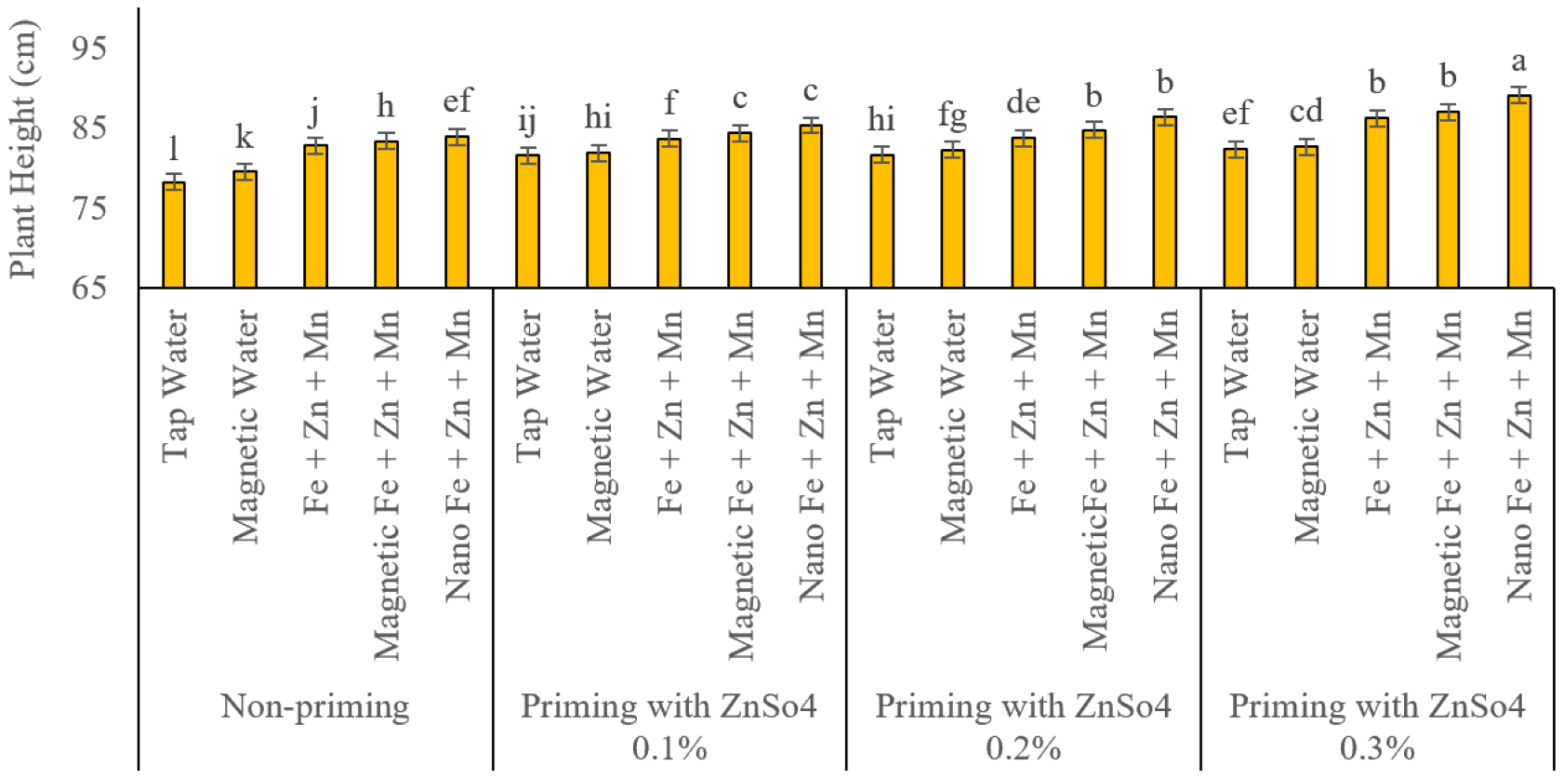
Comparison of the average interaction effects of seed priming with zinc sulphate at different concentrations and foliar spraying of iron, zinc, and manganese (at 2‰), in conventional, magnetic, and nano formulations on time to harvest plant height (day). Means were separated by Least Significant Differences (LSD) at *p* = 0.05; groups sharing at least one letter are not significantly different (*p* > 0.05).

Figure 3 reports the results of plant heights recorded. A significant increase in plant height due to seed priming with zinc sulphate and spraying with micronutrients of iron, zinc and manganese. Based on the results of the comparison of the average interaction effects of the factors of this study, foliar spraying with magnetized treated water significantly increased plant height in all seed priming concentration levels compared to regular water. In addition, among the foliar spraying treatments, the nano and magnetized treatment forms of nutrients had a greater effect on increasing plant height. With increasing zinc sulphate concentration in seed priming treatments, the values of this trait also increased significantly. The lowest and highest plant heights were obtained from the treatment of no seed priming and spraying with regular water and seed priming with zinc sulphate at a concentration of 0.3%, and foliar spraying with iron, zinc and manganese in nano form with plant heights of 27.78 and 1.89 cm, respectively, which were statistically significantly different from other treatments (Fig. 3).

### Spike density per square meter

**Figure 4:**
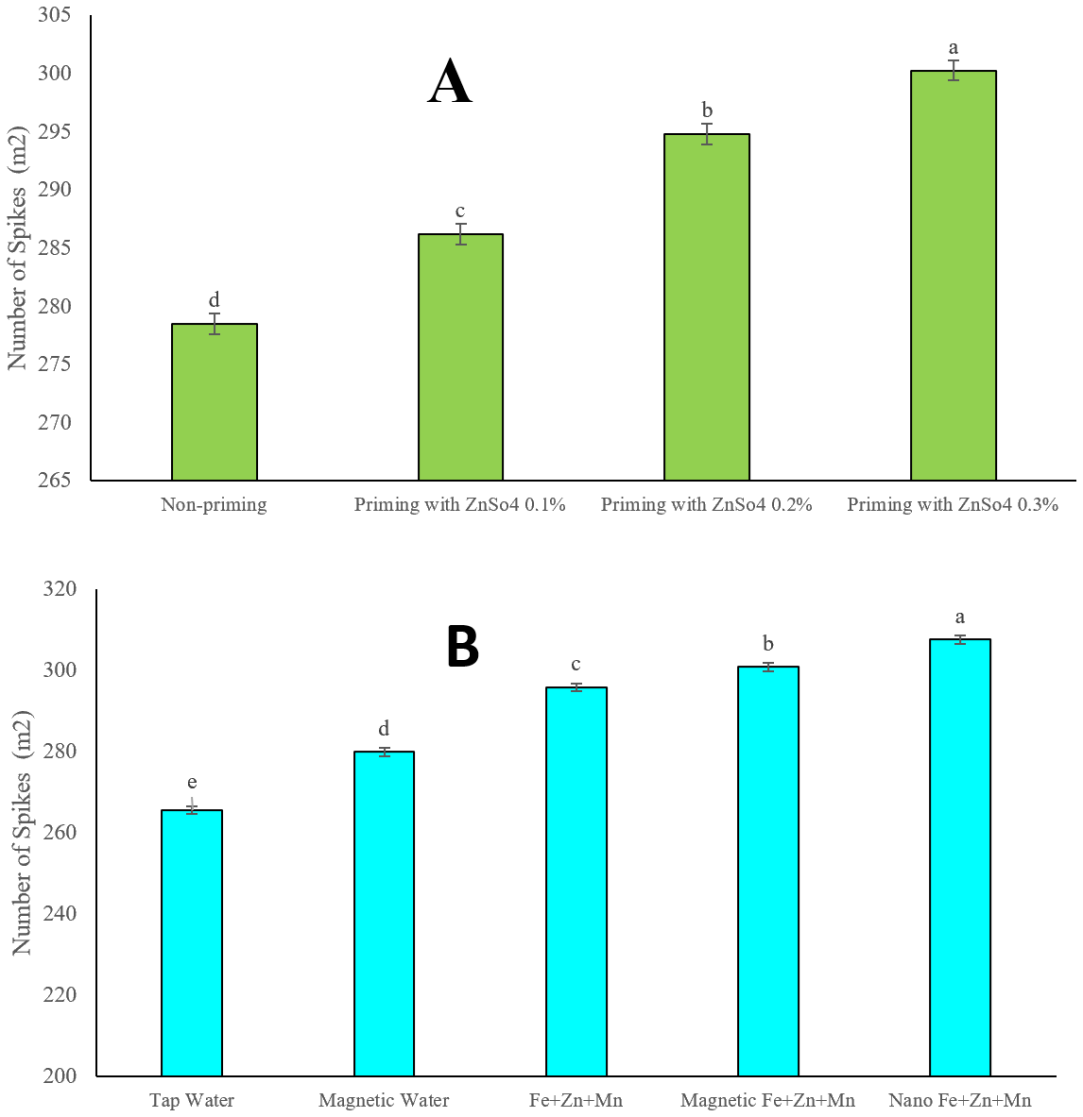
Comparison of the average effect of seed priming with zinc sulfate at different concentrations (A) and foliar spraying with iron, zinc, and manganese in different forms (B) on the number of spikes (m^2^). Means were separated by Least Significant Differences (LSD) at *p* = 0.05; groups sharing at least one letter are not significantly different (*p* > 0.05).

Figure 4 displays the results of spike density per square meter. Given the non-significant interaction effect between seed priming and foliar application on spike density per square meter, treatment comparisons were based on the main effects of each factor. Seed priming with zinc sulphate significantly increased the number of spikes per square meter. Based on mean comparison results, increasing the concentration of zinc sulphate significantly enhanced this key agronomic trait, which plays a crucial role in final yield. The number of spikes per square meter in treatments with no seed priming and seed priming with zinc sulphate at concentrations of 0.1%, 0.2%, and 0.3% were recorded as 278.5, 286.2, 294.8 and 300.25, respectively. These levels were statistically distinct, with all treatments differing significantly.

The mean comparison of the five foliar application treatments also yielded noteworthy results. First, none of the studied levels exhibited statistically similar behaviour, placing them in separate independent groups. Second, magnetised water spray significantly increased spike density per square meter compared to regular water. However, this increase was statistically lower than that observed in all three micronutrient foliar applications (iron, zinc, and manganese).

The highest spike density per square meter (307.56 spikes) was achieved with nano-formulated foliar application of these micronutrients. Following this, the magnetised nutrient solution foliar application resulted in 300.8 spikes per square meter. The conventional foliar application of these three elements recorded 295.8 spikes. Meanwhile, spike densities in magnetised water spray and traditional spray of water treatments were 279.87 and 265.62, respectively.

### Number of seeds per spike

**Figure 5:**
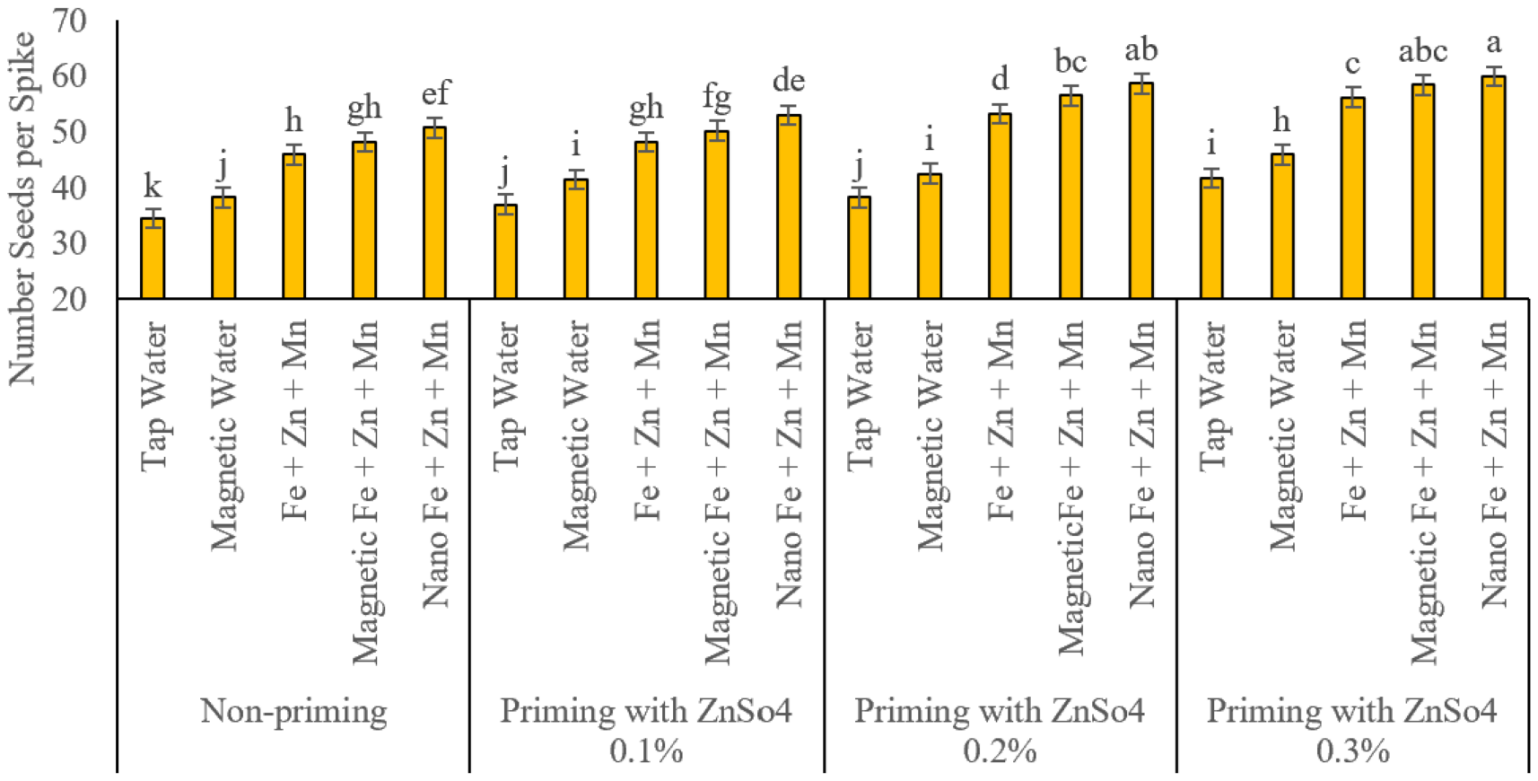
Comparison of the average interaction effects of seed priming with zinc sulphate at different concentrations and foliar spraying of iron, zinc, and manganese (at 2‰), in conventional, magnetic, and nano formulations on number of seeds per spike. Means were separated by Least Significant Differences (LSD) at *p* = 0.05; groups sharing at least one letter are not significantly different (*p* > 0.05).

Figure 5 shows the results of the number of seeds per spike. Given the significant interactive effects of the studied factors on the number of seeds per spike, treatment comparisons were conducted using data from the interaction between seed priming and foliar application. Among the treatment combinations, seed priming with zinc sulphate at concentrations of 2% and 3% and foliar application of iron, zinc, and manganese in nano and magnetised forms increased the number of seeds per spike. The recorded seed counts for these treatments were 60 and 58.5, respectively. Under these two foliar application treatments, in the presence of seed priming with zinc sulphate at a concentration of 0.2%, the number of seeds per spike was recorded as 58.75 and 56.50, respectively. Statistically, these four treatment combinations showed no significant differences. Foliar application of regular and magnetised water at all levels of seed priming resulted in a significantly lower number of seeds per spike. However, magnetised water spraying led to a significant increase in this trait compared to regular water. The lowest number of seeds per spike was observed in the regular water spray treatment without seed priming, with a recorded value of 34.5 seeds.

### Thousand-seed weight

**Figure 6:**
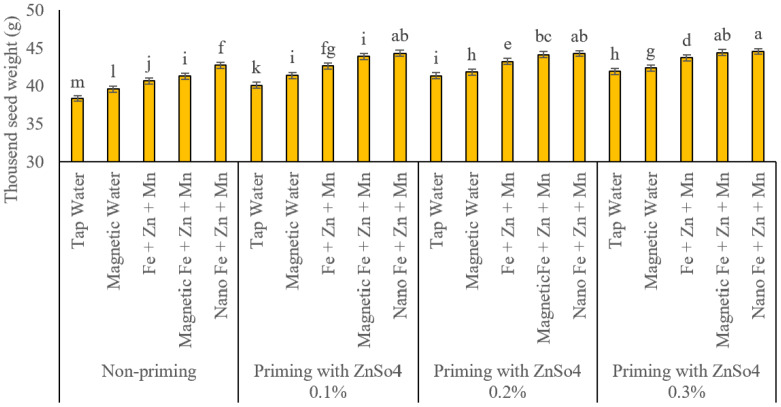
Comparison of the average interaction effects of seed priming with zinc sulphate at different concentrations and foliar spraying of iron, zinc, and manganese (at 2‰), in conventional, magnetic, and nano formulations on thousand seed weight (g). Means were separated by Least Significant Differences (LSD) at *p* = 0.05; groups sharing at least one letter are not significantly different (*p* > 0.05).

Thousand-seed weight, a key determinant of final wheat yield, was significantly influenced by the interaction between seed priming with zinc sulphate (ZnSO_4_) and foliar application of micronutrients (iron, zinc, and manganese) at a concentration of two per thousand in different formulations (Fig. 6). Mean comparison analysis revealed that seed priming and foliar application significantly increased thousand-seed weight. However, among the evaluated treatment combinations, nano-formulated and magnetised foliar applications had a more significant positive impact on thousand-seed weight than conventional foliar applications of these micronutrients. Like other measured traits, magnetised water spray significantly increased thousand-seed weight compared to regular water. The results indicate that increasing zinc sulphate concentration in seed priming significantly enhanced thousand-seed weight. The highest thousand-seed weight was recorded in seeds primed with zinc sulphate at a concentration of 0.3%, while the lowest value (38.35 g) was observed in the regular water spray treatment without seed priming.

### Seed yield

**Figure 7:**
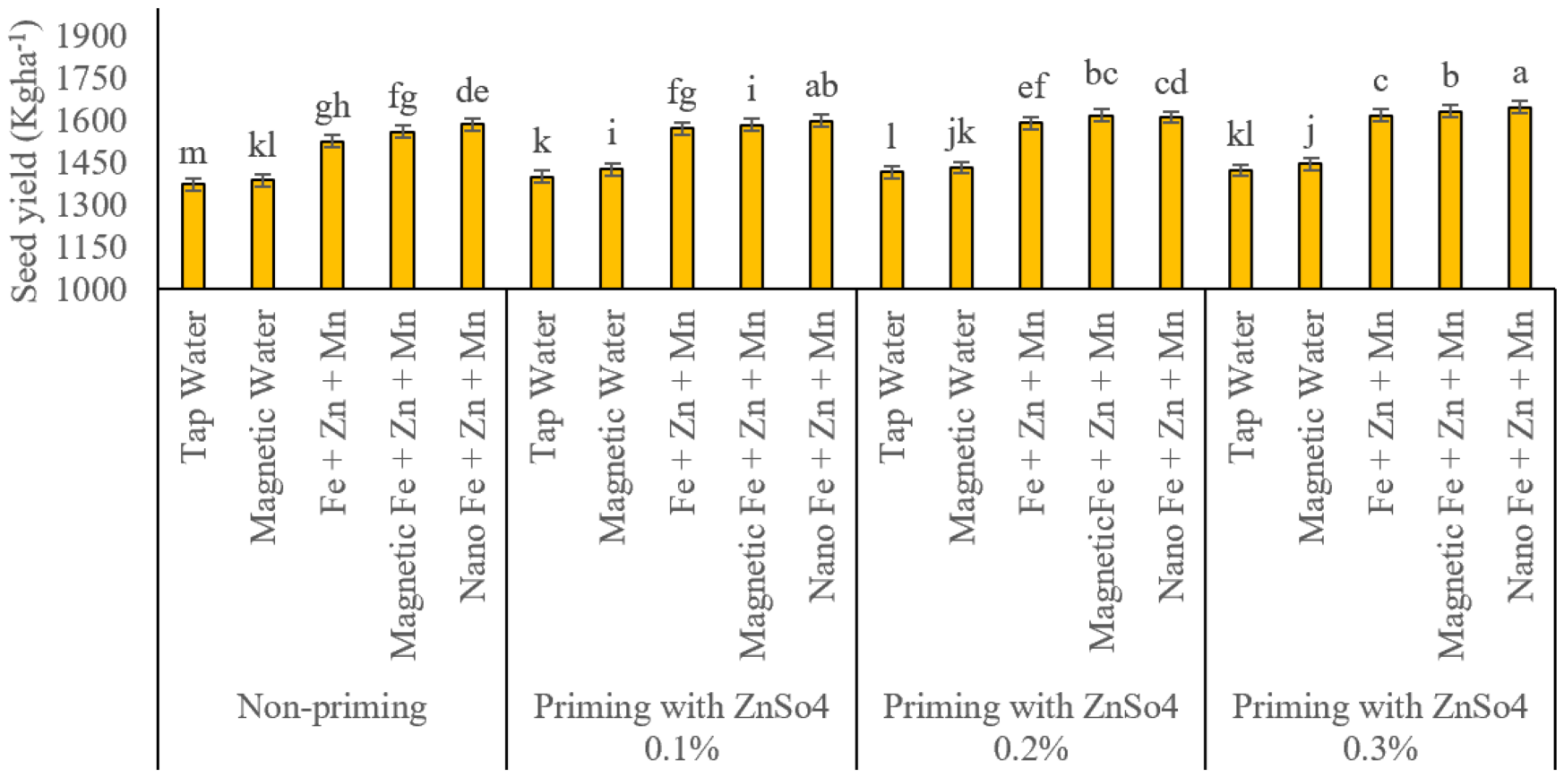
Comparison of the average interaction effects of seed priming with zinc sulphate at different concentrations and foliar spraying of iron, zinc, and manganese (at 2‰), in conventional, magnetic, and nano formulations on seed yield (kg ha^−1^). Means were separated by Least Significant Differences (LSD) at *p* = 0.05; groups sharing at least one letter are not significantly different (*p* > 0.05).

Seed yield was significantly influenced by seed priming with zinc sulphate (ZnSO_4_) and foliar application of iron, zinc, and manganese (Fig. 7). In the absence of seed priming, magnetised water spray and foliar application of these micronutrients in all three formulations significantly improved seed yield compared to regular water spray. However, magnetised water spray had a lesser impact among these treatments than all three micronutrient foliar applications. The lowest seed yield was recorded under no seed priming and regular water spray treatment, with a 1.373 kg ha^−1^ yield. This treatment exhibited the lowest performance among all studied treatments and was statistically classified as an independent group. Magnetised water spray resulted in a seed yield of 1.387 kg ha^−1^. Conventional foliar application of micronutrients produced a significantly higher seed yield (1.526 kg ha^−1^) than control treatments. The nano-formulated foliar application further increased seed yield to 1.560 kg ha^−1^. Statistically, there was no significant difference between conventional and nano-foliar treatments under non-primed conditions. However, the nano-formulation demonstrated the highest positive impact on seed yield, reaching 1.582 kg ha^−1^. Under seed priming with 0.1% ZnSO_4_, all foliar treatments exhibited statistically distinct effects. The recorded seed yields for regular water spray, magnetised water spray, conventional, magnetised, and nano-formulated micronutrient applications were 1.401, 1.427, 1.537, 1.584 and 1.600 kg ha^−1^, respectively. These results indicate that nano-foliar application had the greatest impact on seed yield, followed by the magnetised nutrient application. A similar trend was observed under seed priming with 0.2% ZnSO_4_. The only difference was that nano and magnetised foliar applications of iron, zinc, and manganese exhibited statistically identical effects, yielding 1.619 and 1.613 kg ha^−1^, respectively. Additionally, conventional foliar application of these elements resulted in a seed yield of 1.593 kg ha^−1^, while magnetised and regular water sprays produced 1.433 and 1.417 kg ha^−1^, respectively. At the highest seed priming level (0.3% ZnSO_4_), all foliar application treatments had statistically distinct effects on seed yield. The highest recorded yield (1.648 kg ha^−1^) was obtained from nano-foliar application of micronutrients. This was followed by magnetised foliar application (1.632 kg ha^−1^) and conventional micronutrient foliar application (1.618 kg ha^−1^). Statistically, the conventional foliar treatment yielded significantly lower results than the nano and magnetised formulations. Under these conditions, magnetised and regular water sprays produced seed yields of 1.446 and 1.423 kg ha^−1^, respectively.

### Biological yield

**Figure 8:**
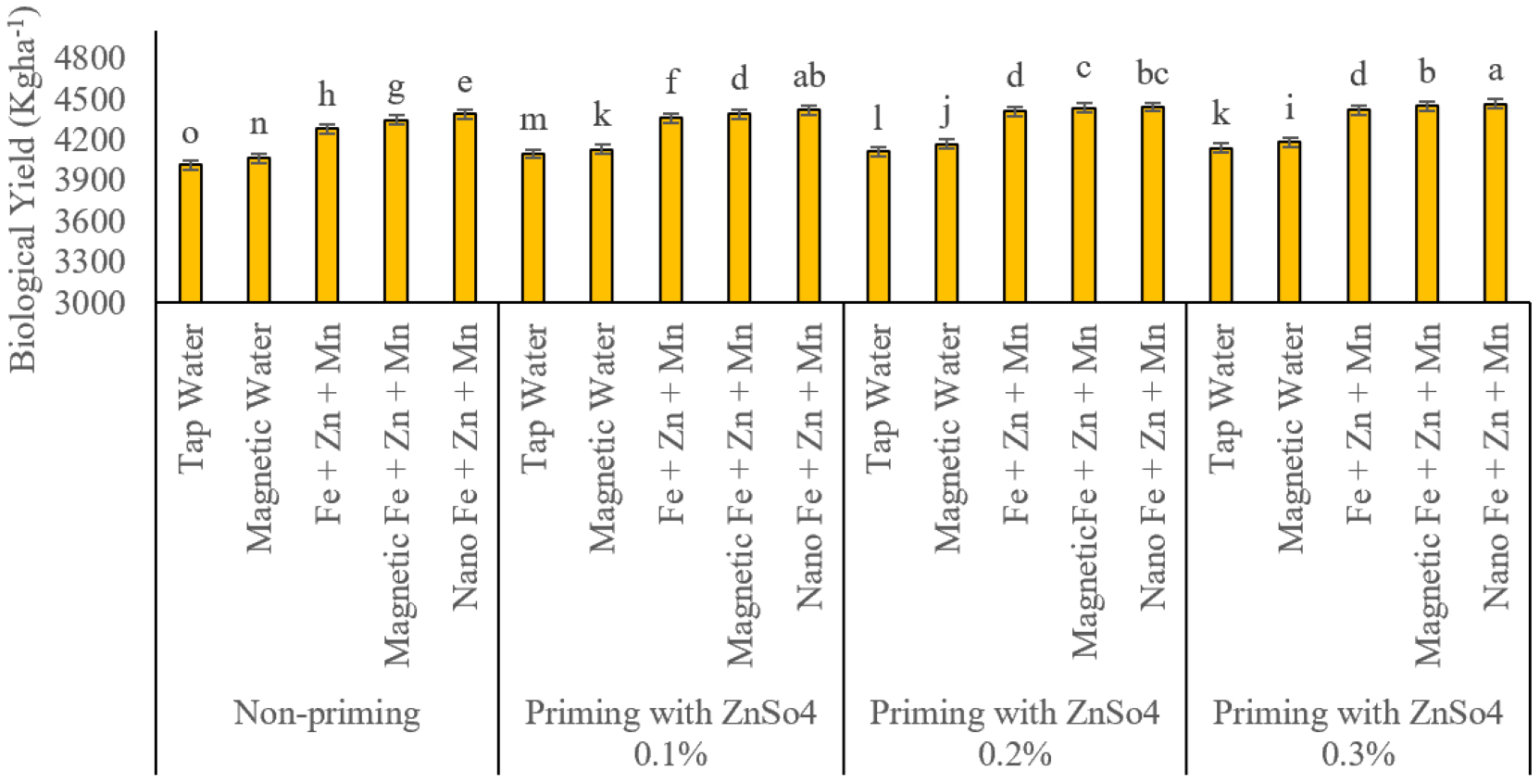
Comparison of the average interaction effects of seed priming with zinc sulphate at different concentrations and foliar spraying of iron, zinc, and manganese (at 2‰), in conventional, magnetic, and nano formulations on biological yield (kg ha^−1^). Means were separated by Least Significant Differences (LSD) at *p* = 0.05; groups sharing at least one letter are not significantly different (*p* > 0.05).

In all four seed priming treatments with zinc sulphate (ZnSO_4_), foliar application of iron, zinc, and manganese in nano-formulations significantly increased biological yield (Fig. 8).

The recorded values for this trait under the respective treatments were 4.388, 4.417, 4.441, and 4.465 kg ha^−1^. Magnetized treatment foliar application demonstrated a more significant impact on enhancing biological yield than other treatments among all seed priming levels. Although conventional foliar application of these nutrients also resulted in a statistically significant improvement relative to the control, their effect was less pronounced than that of nano and magnetized treatments. The lowest biological yield (4.014 kg ha^−1^) was observed in the combination of non-primed seeds and conventional water spray.

### Protein percentage and yield

**Figure 9:**
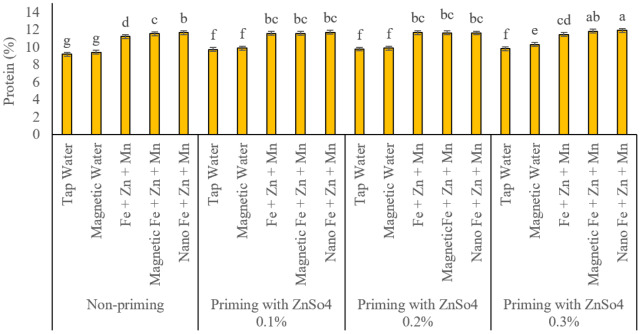
Comparison of the average interaction effects of seed priming with zinc sulphate at different concentrations and foliar spraying of iron, zinc, and manganese (at 2‰), in conventional, magnetic, and nano formulations on protein (%). Means were separated by Least Significant Differences (LSD) at *p* = 0.05; groups sharing at least one letter are not significantly different (*p* > 0.05).

As displayed in Fig. 9, protein percentage increased significantly across all seed priming levels with foliar application of iron, zinc, and manganese in conventional, subjected to a magnetic field, and nano-formulations compared to control treatments (traditional and magnetized treatment water sprays).

**Figure 10:**
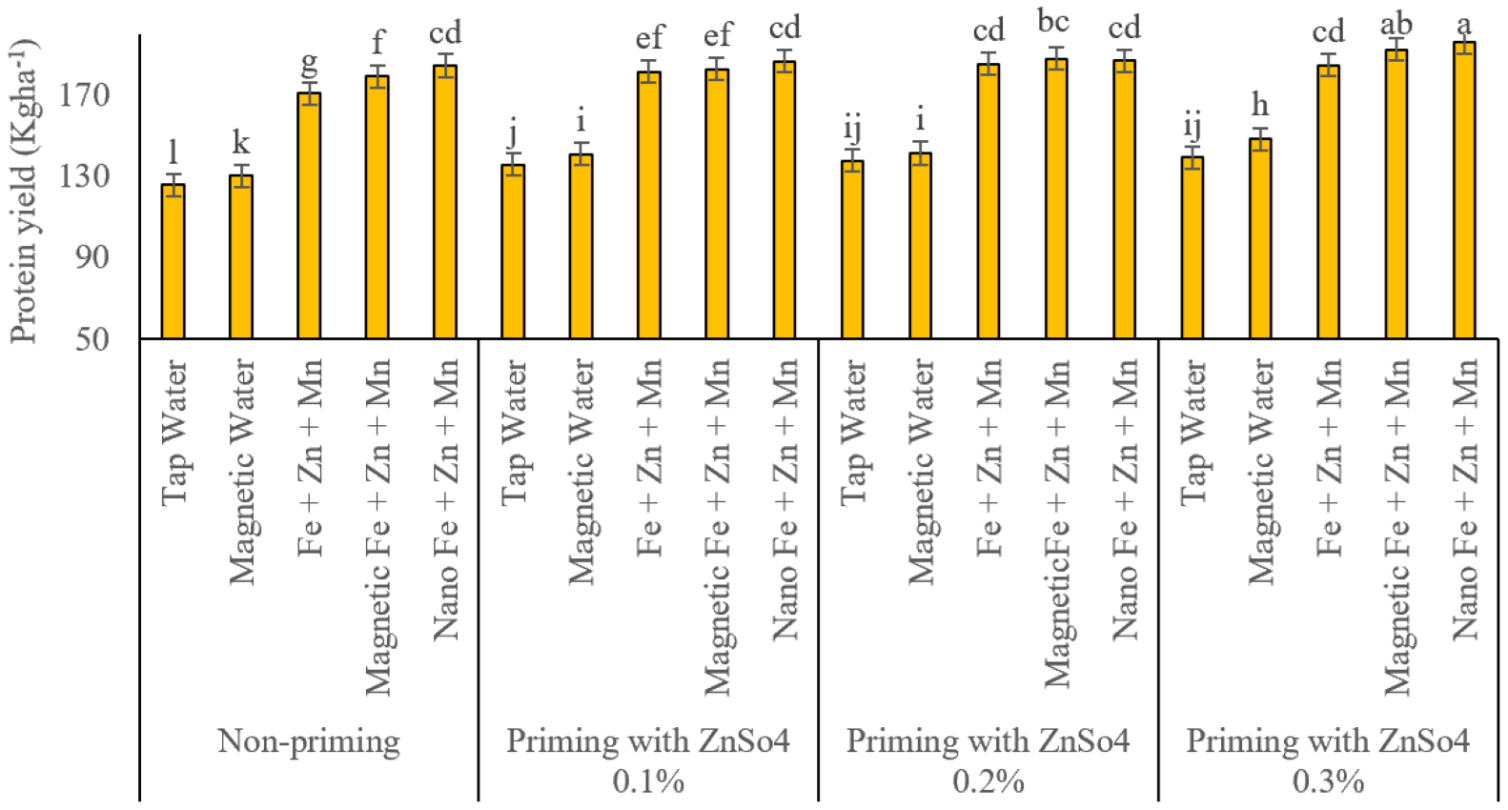
Comparison of the average interaction effects of seed priming with zinc sulphate at different concentrations and foliar spraying of iron, zinc, and manganese (at 2‰), in conventional, magnetic, and nano formulations on protein yield (kg ha^−1^). Means were separated by Least Significant Differences (LSD) at *p* = 0.05; groups sharing at least one letter are not significantly different (*p* > 0.05).

Without seed priming, none of the three foliar nutrient application methods had statistically equivalent effects on protein percentage. Nano, subjected to a magnetic field, and conventional foliar applications resulted in protein percentages of 11.65%, 11.50%, and 11.20%, respectively, while traditional and magnetized treatment water sprays yielded 9.17% and 9.40%, respectively. Unlike previous traits, these two control treatments showed no significant statistical difference. All three foliar nutrient application methods significantly increased protein percentage compared to controls at seed priming concentrations of 0.1% and 0.2% zinc sulphate (ZnSO_4_). However, no significant differences were observed among the foliar methods, nor between conventional and magnetized treatment water sprays under these conditions. In contrast, at the 0.3% ZnSO_4_ priming level, nano and magnetized treatment foliar applications significantly outperformed conventional methods, yielding protein percentages of 11.90%, 11.80%, and 11.43%, respectively. Notably, magnetized treatment and nano treatments had equivalent effects on protein percentage at this priming level. In this scenario, magnetized treatment water spray (10.27%) significantly outperformed conventional water spray (9.80%). Figure 10 displays the protein yield. This parameter was also influenced by seed priming and foliar application methods. Without seed priming, protein yields for conventional water spray, magnetized treatment water spray, and traditional, subjected to a magnetic field, and nano foliar nutrient applications were 125.88, 130.37, 170.99, 179.43, and 184.76 kg ha^−1^, respectively. Statistically, all treatments differed significantly, with progressively increasing effects. At the 0.1% ZnSO_4_ priming level, protein yields for the same treatments were 135.89, 140.96, 181.70, 182.94 and 186.80 kg ha^−1^. Conventional and magnetized treatment applications had similar effects, while nano applications significantly increased protein yield. At the 0.2% ZnSO_4_ priming level, protein yields shifted to 137.87, 141.53, 185.67, 188.23, and 187.15 kg ha^−1^. Both water sprays and all three foliar nutrient treatments exhibited statistically equivalent effects. At the 0.3% priming level, the highest protein yields were observed in nano (196.08 kg ha^−1^) and magnetized treatment(192.63 kg ha^−1^) foliar applications, which were statistically comparable. Conventional foliar application yielded 184.84 kg ha^−1^, significantly higher than controls but lower than nano and magnetized treatment methods. This method (130.37 kg ha^−1^) outperformed conventional water spray (125.88 kg ha^−1^) under these conditions.

### Harvest index

**Figure 11:**
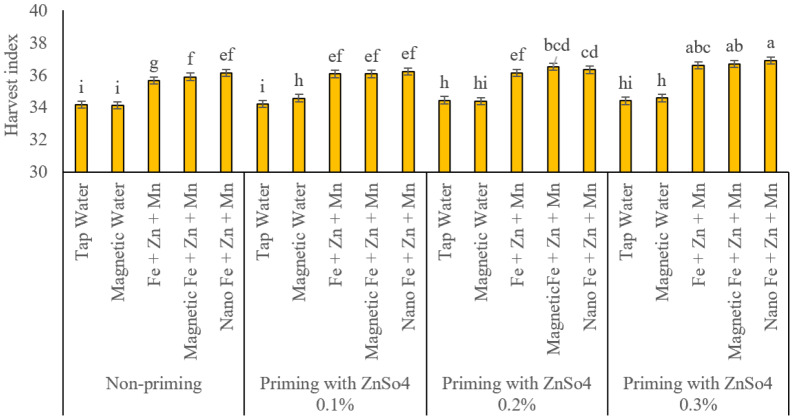
Comparison of the average interaction effects of seed priming with zinc sulphate at different concentrations and foliar spraying of iron, zinc, and manganese (at 2‰), in conventional, magnetic, and nano formulations on the harvest index. Means were separated by Least Significant Differences (LSD) at *p* = 0.05; groups sharing at least one letter are not significantly different (*p* > 0.05).

Figure 11 displays the results of harvest indexes. In the absence of seed priming, the harvest indices for conventional water spray, magnetized treatment water spray, and conventional, subjected to a magnetic field, and nano foliar applications of iron, zinc, and manganese were 34.18%, 34.12%, 35.65%, 35.89%, and 36.14%, respectively. At the 0.1% ZnSO_4_ seed priming level, harvest index values for these treatments were 34.20%, 34.57%, 36.07%, and 36.22%, with no significant differences observed among foliar nutrient application methods. At the 0.2% ZnSO_4_ priming level, harvest index values for conventional and magnetized treatment water sprays were 34.44% and 34.38%, respectively, showing statistically equivalent effects. Conversely, conventional, subjected to a magnetic field, and nano foliar applications of the nutrients yielded harvest index values of 36.12%, 36.52%, and 36.33%, respectively. Magnetized treatment and nano foliar treatments significantly outperformed conventional methods in enhancing harvest index under these conditions. At the 0.3% ZnSO_4_ priming level, conventional, subjected to a magnetic field, and nano-foliar applications exhibited comparable effects on HI, with recorded values of 36.59%, 36.69%, and 36.91%, respectively. Conventional and magnetized treatment water sprays also demonstrated similar impacts, with a harvest index of 34.40% and 34.57%.

### Data modelling

**Figure 12:**
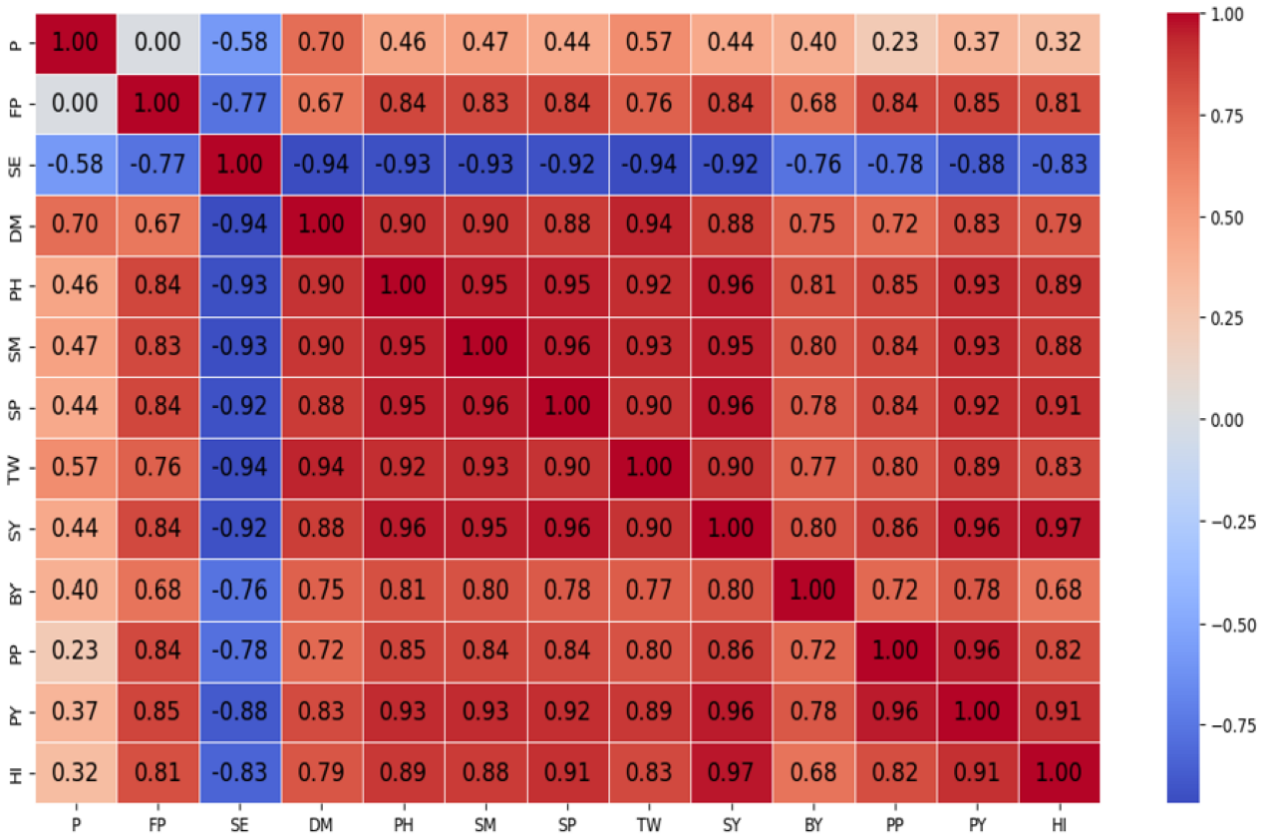
Correlation coefficients obtained from data modelling of the studied traits (SE, Spike Emergence; DM, Day to Harvest; PH, Plant Height; SM, Number of Spikes; SP, Number Seeds per Spike; TW, Thousand seed Weight; SY, Seed Yield; BY, Biological Yield; PP, Protein (%), Protein yield (kg ha^−1^); HI, Harvest index. The colors range from red, representing a strong positive correlation, to blue, indicating a strong negative correlation, with gray denoting no correlation.

Table S1 and Fig. 12 report the results obtained from data modelling.

The analysis of various machine learning models reveals distinct performance differences in predicting key agronomic traits of wheat. Ridge, Lasso, and EN demonstrated the highest predictive accuracy among the tested algorithms, particularly for biological yield, protein content, protein yield, and harvest index, achieving R^2^ values close to 0.99. These models also exhibited the lowest RMSPE across training and test datasets, indicating strong generalisation capabilities. The superior performance of these models is attributed to their ability to mitigate overfitting through regularisation, ensuring a balanced trade-off between model complexity and predictive accuracy.

Boosting algorithms, specifically XGB and CB, emerged as highly effective models, particularly for predicting biological yield, protein content, and harvest index, with R^2^ values nearing 0.99 and minimal RMSPE in the test data. These models excel at capturing complex nonlinear interactions among variables, contributing to their high accuracy.

RF and SVR displayed inconsistent performance across different traits. While RF performed well in predicting Spike Emergence and number of spikes per meter, it exhibited significant errors in predicting Biological yield and protein yield, as reflected in the elevated RMSPE values for test data. Similarly, SVR performed adequately for seed yield and protein yield but struggled with biological yield and thousand seed weight, indicating potential challenges related to feature scaling and hyperparameter optimisation.

Overall, Ridge, Lasso, and EN emerged as the most reliable models for predicting linear relationships and highly correlated traits, while XG and CB proved superior in handling complex nonlinear interactions. Random Forest and SVR showed promising results in specific cases but exhibited greater variability in performance across different traits. Future studies should focus on optimising XGB and CB hyperparameters for improved stability, while Ridge and Lasso remain the preferred choices for ensuring robust predictive accuracy in agronomic modelling. Overall, the combination of careful preprocessing, robust feature selection, and hyperparameter tuning enables reliable predictions across most traits, providing a methodological framework adaptable to other crops and environmental conditions.

### SHAP feature attribution

**Figure 13:**
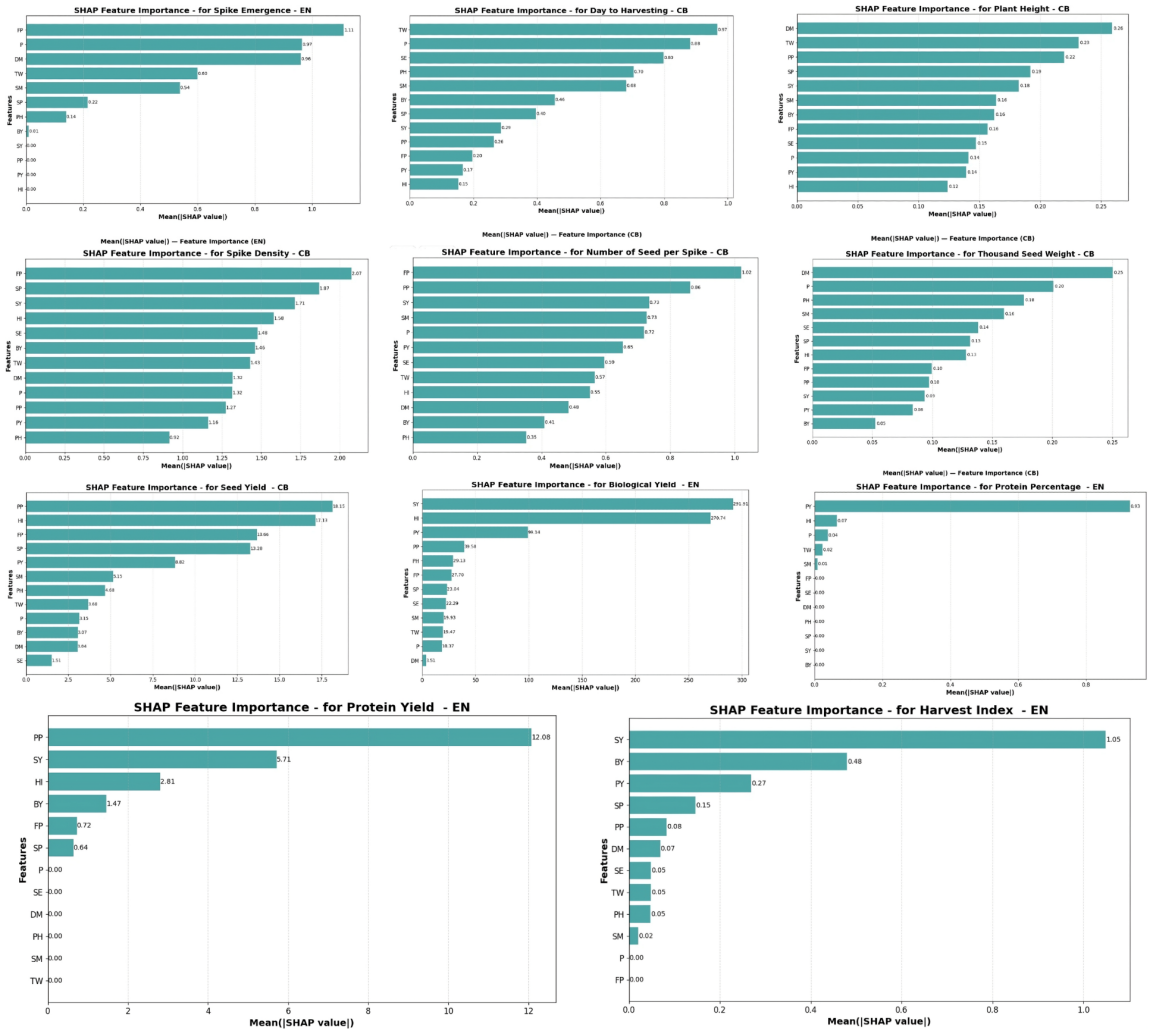
SHAP Feature Importance of the best models for all the studied traits (SE, Spike Emergence; DM, Day to Harvest; PH, Plant Height; SM, Number of Spikes; SP, Number Seeds per Spike; TW, Thousand seed Weight; SY, Seed Yield; BY, Biological Yield; PP, Protein (%), Protein yield (kg ha^−1^); HI, Harvest index.

SHAP analyses were performed for every trait across all regression models evaluated (see Supplementary Material). We highlight the best-performing models—CB and EN—and report their results in detail in Fig. 13. The results show that distinct patterns of feature contribution depend on both the target trait and the model type. Longer bars on the plots suggest features with higher predictive impact, corresponding to high values of the mean absolute SHAP values. For spike emergence, the EN model indicated that day to harvesting, thousand seed weight, and spike number had the greatest effects, with mean SHAP values of 0.96, 0.60, and 0.54, respectively. Using the CatBoost (CB) model for Days to Harvesting, the variables thousand seed weight, spike emergence, and number of spikes showed the highest mean SHAP values (0.97, 0.80, and 0.68).

For the morphological characters, *i.e.,* plant height and spike density, CatBoost was again the most interpretable model. Plant Height was mainly affected by Day to Harvesting, thousand seed weight, and protein percentage, whereas spike density was strongly influenced by number of seeds per spike, seed yield, and harvest index, revealing a close correlation between structural and reproductive components. Likewise, number of seeds per spike, thousand seed weight, and seed yield were best explained using CatBoost.

For the remaining traits such as biological yield, protein percentage, protein yield, and Harvest Index, the EN model provided the highest interpretability results. For Biological yield, the highest mean SHAP values were observed for seed yield (291.91), harvest index (270.74), and protein yield (99.14), reflecting both the large scale of the target variable and the highly interconnected nature of biomass-related traits. Protein percentage was primarily impacted by protein yield (0.93). Meanwhile, protein yield was strongly influenced by protein percentage (12.08), Seed Yield (5.71), and harvest index (2.81). Finally, for harvest index, the Elastic Net model identified seed yield, biological yield, and protein yield as the strongest predictors, with corresponding SHAP values of 1.05, 0.48, and 0.27, respectively. The scale of the SHAP values is directly proportional to the range of scale and sensitivity of model predictions with respect to the input features. Properties such as biological yield and seed Yield inherently have large number ranges, thus contributing proportionally large values of SHAP. On the other hand, features like spike emergence or protein percentage have measurements on lesser numeric ranges, hence their SHAP magnitudes stay lesser.

Tree-based models have a propensity for smaller, smoother SHAP magnitudes due to their predictions being constructed out of averaged decision paths.

Conversely, linear (particularly EN) models produce high values for SHAP if coefficients are not standardized or if there are correlations between predictors, because the SHAP values linearly scale with model coefficients and variances of features. Interrelated features such as Spike Yield and Biological Yield both have high of SHAP magnitudes, which indicate their mutual impact on yield-related features.

In general, the analysis by SHAP showed the highly yield-associated traits (especially SY, SP, and BY) were the strongest predictors for the majority of the responses under investigation.

Moreover, the application of SHAP gave the model transparency, which provided quantitative explanation of both feature importance and feature interdependence of traits, which were not easily interpreted using traditional regression coefficients or correlation analysis.

## Discussion

Rainfed wheat cultivation in arid and semi-arid regions faces numerous challenges, including limited moisture and nutrient deficiencies (Tadesse & Asefa, 2025).

Seed priming, by increasing the activity of hydrolytic enzymes such as alpha-amylase, protease, and lipase, leads to faster breakdown of seed reserves and provides the energy needed to start growth. As a result, primed seeds have higher germination speed and uniformity, which leads to increased density and uniformity of rain-fed fields. Also, in irregular rainfall conditions, plants with better initial growth will have a better chance of utilising limited soil moisture and will be more resistant to environmental stresses (Moradi & Siosemardeh, 2023).

Physiologically, seed priming increases root development and improves water and nutrient uptake in the early stages of growth. Seed priming with micronutrients like zinc sulphate enhances auxin synthesis, promoting longer and denser roots that improve water uptake and use efficiency under rainfed conditions, leading to greater leaf area, photosynthesis, and grain yield (kalyani et al., 2022).

As Rizk and collaborators found, seed priming with zinc sulphate is an efficient solutions to improve wheat cultivation in rainfed conditions and it is considered one of the low-cost treatment. In such situations, a lack of moisture and nutrients often prevents proper seedling establishment and optimal growth, and as a result, crop yield is severely reduced (Rizk et al., 2025). Seed priming with zinc enhances hydrolytic enzyme activity, cell division, and root growth, accelerating germination and improving water and nutrient absorption (Kaur et al., 2024). Zinc also reduces oxidative stress during drought, boosts carbonic anhydrase activity, and enhances CO_2_ fixation, leading to increased biological growth and grain yield in rainfed wheat (Singhal, Pandey & Bose, 2021).

In addition to seed priming, foliar application of micronutrients also significantly strengthens wheat nutrition in rainfed conditions. For example, Helmy et al. compared the impact of magnetized water (MW) and magnetized seed treatment (MS) on wheat productivity grown in salty soil. They described that the combination of compost and magnetic treatments resulted in a large increase in chlorophyll content (up to 189% increase) and carbohydrate accumulation (220% increase). It was found that the maximum benefits in grain yield (maximum of 846 percent) and spike weight (maximum of 845 percent) were found when compost was used in combination with magnetic treatments, which demonstrate the synergistic effects of these treatment on the productivity of wheat (Helmy et al., 2023). These results are consistent with those observed in this study, where nano- and magnetized foliar treatments were found to enhance grain protein content and the uptake of micronutrients, such as Zn and Fe. This suggests that the combination of seed priming with advanced foliar treatments has considerable potential for improving wheat productivity, especially in resource-limited environments. The simultaneous combination of seed priming and micronutrient foliar application showed the greatest effectiveness in the key indicators of rainfed wheat growth and yield. This combination increases the rate of spike emergence, the number of tillers per square meter and the number of grains per spike, ultimately leading to improved grain yield (Ahmad et al., 2024).

Regarding quality, priming with zinc and foliar application of micronutrients, especially in nano and magnetized treatment form, also increased the percentage of grain protein and protein yield per hectare. By increasing the activity of nitrate reductase and glutamine synthetase enzymes, these elements act towards nitrogen fixation and the synthesis of amino acids. Considering these findings, applying these methods can be recommended for developing sustainable agriculture in arid and semi-arid regions (Verma et al., 2023). In addition to the agronomic treatments discussed, the use of magnetic water has shown promise in improving plant growth under stressful conditions, enhancing nutrient absorption and increasing overall yield. Magnetic water can modify the physicochemical properties of the solution, lowering surface tension and increasing ion mobility, which boosts foliar penetration and photosynthesis under drought conditions. While the physiological effects of biostimulant treatments are well-established, predicting their impact in complex agricultural environments requires sophisticated modeling tools. This is where machine learning (ML) models, such as SVR, RF, and XGB, become invaluable. This research underscores the importance of choosing the most appropriate machine learning models to accurately predict key agronomic traits in wheat, while also acknowledging several limitations that should be considered. First, the field trial was conducted at a single location and in a single season, which restricts the generalizability of the findings. Rainfed wheat systems are highly influenced by inter-annual climatic variability, and multi-year trials are necessary to validate the consistency of the observed results. Second, the magnetized water treatment, although promising, still lacks standardised protocols and may produce variable outcomes depending on the equipment used. Finally, while machine learning provided valuable predictive insights, the dataset size was relatively limited, which may reduce robustness for certain traits. Broader datasets from diverse environments and growing conditions are needed for model refinement. Moreover, also the economic feasibility is also an essential aspect. While nano-fertilizers and magnetization equipment demonstrated beneficial effects, their costs may be prohibitive in low-income or smallholder farming systems. Therefore, before broad adoption, cost-benefit analyses should be conducted, considering not only yield and protein improvements but also the accessibility of such technologies for farmers. In many cases, conventional or low-cost seed priming strategies may remain the most realistic option, while nano- and magnetized formulations could be prioritised in high-value or resource-supported production systems. The superior performance of Ridge, Lasso, and Elastic Net in traits such as biological yield, protein content, protein yield, and harvest index suggests that regularised regression methods effectively capture linear relationships and mitigate overfitting, making them highly suitable for datasets with multicollinearity. These models provide stable and interpretable predictions essential for agronomic decision-making. Conversely, tree-based and boosting models generally outperformed others because they naturally capture nonlinear relationships and interactions between input features, which are prevalent in agronomic datasets. XGB and CB accurately captured complex, nonlinear patterns, particularly in thousand see weight, biological yield, protein content, and harvest index. Their ability to model interactions and hierarchical dependencies contributed to their high predictive power. Their robustness to correlated features and outliers further enhances generalization. Linear models performed reliably for traits with predominantly linear relationships, serving as interpretable baselines. In our study, SHAP analysis revealed model-specific attribution patterns: tree-based models produced smaller, smoother contributions due to averaging across decision paths, whereas linear models—especially Elastic Net—showed larger values when coefficients were unstandardized or predictors were correlated. Yield-related traits (SY, SP, BY) consistently had the highest SHAP values, marking them as dominant predictors. Beyond ranking features, SHAP clarified interdependence among correlated variables—insights not readily captured by regression coefficients or simple correlations. Their ability to model interactions and hierarchical dependencies contributed to their high predictive power. This insight can guide agronomists in prioritizing field measurements and optimizing fertilization or biostimulant schedules with minimal data input. However, their performance in predicting the Number of seeds per spike and thousand-seed weight was less consistent, likely due to sensitivity to hyperparameter selection. It is important to note that model performance was not uniform across all traits. Specifically, the prediction of seed number and thousand-seed weight (TSW) was less accurate than for other variables. This reduced performance may stem from the higher sensitivity of these traits to environmental fluctuations, measurement variability, and limited representation in the dataset. Additionally, tree-based algorithms such as XGB and CatBoost may have required further hyperparameter optimisation to improve stability in predicting seed-related features. Future research should therefore focus on larger datasets, cross-seasonal training, and fine-tuned model calibration to enhance predictions for these critical yield components. RF and SVR exhibited more variability in performance. In fact, RF excelled in specific traits such as spike emergence and number of spikes per meter but showed higher error rates in predicting Biological Yield and protein Yield. RF’s limitations could be attributed to its sensitivity to feature importance and the curse of dimensionality. At the same time, SVR’s performance fluctuations may result from kernel selection and scaling issues. Choosing an optimal machine learning model depends on the complexity of the trait being predicted (Chlingaryan, Sukkarieh & Whelan, 2018). Regularised regression methods remain the best option for traits with strong linear relationships, while tree-based ensemble models such as XGB and CB offer superior performance for nonlinear relationships. To further enhance predictive accuracy, future research should explore hyperparameter optimisation techniques, feature engineering strategies, and integrate deep learning approaches for agronomic modelling.

## Conclusions

The findings of this study demonstrate that seed priming with ZnSO_4_ and foliar application of micronutrients in advanced forms (nano and subjected to a magnetic field) significantly enhance rainfed wheat performance. Seed priming accelerated spike emergence, improved plant height, and increased biological yield, particularly at higher ZnSO_4_ concentrations. Foliar application further enhanced yield components, with nano and magnetized treatments proving superior to conventional applications. These treatments increased the number of spikes per square meter, seeds per spike, and the thousand-seed weight, ultimately improving grain yield and protein content. The highest grain yield was recorded under 0.3% ZnSO_4_ seed priming combined with nano-foliar application, indicating a synergistic effect. Additionally, the study employed machine learning models to predict agronomic traits, with Ridge, Lasso, and EN performing best for linear relationships, while XGB and CB effectively modelled complex nonlinear interactions. However, these models showed variability in predicting specific seed-related traits, suggesting that further optimisation is needed. Overall, the combination of seed priming and advanced foliar application methods offers an effective approach to increasing the resilience and productivity of rainfed wheat. Future research should explore the long-term effects of these treatments, their economic feasibility, and potential applications in other crops under varying environmental conditions.

##  Supplemental Information

10.7717/peerj.20578/supp-1 Supplemental Information 1 Results of data modeling using different machine learning methods SE: Spike emergence (day), DM: Day to Harvesting, PH: Plant height, SM: Number of Spikes (m2), SP: Number Seeds per spike, TW: Thousand seed weight (g), SY: Seed yield (kg ha^−1^), BY: Biological Yield (kg ha^−1^), PP: Protein (%), Protein yield (kg ha^−1^), HI: Harvest index.

10.7717/peerj.20578/supp-2 Supplemental Information 2 Hyperparameter Ranges Used for Model Tuning

10.7717/peerj.20578/supp-3 Supplemental Information 3 SHAP Feature Importance of the models for all the studied traits

10.7717/peerj.20578/supp-4 Supplemental Information 4 Spike emergency day, time to harvest, plant height, number of spikes, seed yield, biological yield, protein content, and harvest index The measurement for each variable includes, like Spike Emergency (day), Time to Harvest (day), Plant Height (cm), Number of Spikes (m2), Number Seeds per Spike, Thousand seed weight (g), Seed yield (Kgha-1), Biological Yield (Kgha-1), Protein (%), Protein yield (Kgha-1), Harvest index
Spike Emergency (day), Time to Harvest (day), Plant Height (cm), Number of Spikes (m2), Number Seeds per Spike Thousand seed weight (g), Seed yield (Kgha-1), Biological Yield (Kgha-1), Protein (%), Protein yield (Kgha-1), Harvest index).

10.7717/peerj.20578/supp-5 Supplemental Information 5 Code for ML in Rainfed Wheat: Seed Priming Effects

## Additional Information and Declarations

### Data Availability

The following information was supplied regarding data availability: The raw data and code are available in the Supplemental Files. Data is also available at Zenodo: Talebian, B., Ghiyasi, M., & Sharifi, L. (2025). RainfedWheat [Data set]. Zenodo. https://doi.org/10.5281/zenodo.17800091
The code is available at Kaggle and Zenodo: https://www.kaggle.com/code/bardiatalebian/ml-in-rainfed-wheat-seed-priming-effects
Talebian, B. (2025). ML in Rainfed Wheat: Seed Priming Effects. Zenodo. https://doi.org/10.5281/zenodo.17800708.
