# Supplementary material for: Machine learning-based evaluation of seed priming and biostimulant applications in rainfed wheat
Source: PeerJ. 2026 Mar 2;14:e20578. doi: 10.7717/peerj.20578 (PMC12962134; doi:10.7717/peerj.20578)
Supplement: Supplemental Information 1 — SE: Spike emergence (day), DM: Day to Harvesting, PH: Plant height, SM: Number of Spikes (m2), SP: Number Seeds per spike, TW: Thousand seed weight (g), SY: Seed yield (kg ha−1), BY: Biological Yield (kg ha−1), PP: Protein (%), Protein yield (kg ha−1), HI: Harvest index. [file peerj-14-20578-s001.docx]

| **R^2^ (Test)** | **R^2^ (Train)** | **RMSPE (Test)** | **RMSPE (Train)** | **R^2^ (Test)** | **R^2^ (Train)** | **RMSPE (Test)** | **RMSPE (Train)** | **Model** |
| --- | --- | --- | --- | --- | --- | --- | --- | --- |
|  |  | **DM** |  |  | **SE** |  |  |  |
| 0.93 | 0.95 | 0.86 | 0.71 | 0.89 | 0.90 | 1.13 | 1.01 | Linear |
| 0.94 | 0.96 | 0.84 | 0.60 | 0.90 | 0.91 | 1.07 | 0.97 | Ridge |
| 0.93 | 0.96 | 0.79 | 0.60 | 0.87 | 0.90 | 1.24 | 1.03 | Lasso |
| 0.93 | 0.97 | 0.84 | 0.57 | 0.91 | 0.92 | 1.02 | 0.92 | EN |
| 0.91 | 0.96 | 0.91 | 0.61 | 0.91 | 0.92 | 1.03 | 0.91 | SVR |
| 0.87 | 0.95 | 1.13 | 0.73 | 0.87 | 0.94 | 1.27 | 0.76 | RF |
| 0.85 | 0.99 | 1.21 | 0.002 | 0.88 | 0.99 | 1.23 | 0.24 | XGB |
| 0.94 | 0.99 | 0.80 | 0.14 | 0.86 | 0.99 | 1.30 | 0.17 | CB |
|  |  | **SM** |  |  |  | **PH** |  |  |
| 0.92 | 0.93 | 1.82 | 1.56 | 0.90 | 0.94 | 0.90 | 0.72 | Linear |
| 0.93 | 0.93 | 1.72 | 1.55 | 0.89 | 0.94 | 0.91 | 0.72 | Ridge |
| 0.92 | 0.93 | 1.72 | 1.53 | 0.89 | 0.94 | 0.96 | 0.70 | Lasso |
| 0.92 | 0.92 | 1.83 | 1.66 | 0.90 | 0.94 | 0.94 | 0.69 | EN |
| 0.94 | 0.92 | 1.57 | 1.59 | 0.90 | 0.99 | 1.01 | 0.11 | SVR |
| 0.93 | 0.95 | 1.63 | 1.26 | 0.90 | 0.96 | 0.90 | 0.56 | RF |
| 0.94 | 1.00 | 1.47 | 0.0001 | 0.90 | 0.99 | 0.94 | 0.009 | XGB |
| 0.95 | 0.99 | 1.38 | 0.25 | 0.90 | 0.99 | 0.90 | 0.14 | CB |
|  |  | **TW** |  |  |  | **SP** |  |  |
| 0.90 | 0.91 | 1.33 | 1.20 | 0.93 | 0.95 | 4.25 | 3.62 | Linear |
| 0.91 | 0.92 | 1.21 | 0.99 | 0.92 | 0.96 | 4.63 | 3.03 | Ridge |
| 0.93 | 0.93 | 1.07 | 1.04 | 0.92 | 0.96 | 4.60 | 3.02 | Lasso |
| 0.92 | 0.93 | 1.11 | 0.98 | 0.92 | 0.96 | 4.64 | 3.04 | EN |
| 0.92 | 0.99 | 1.14 | 0.40 | 0.86 | 0.96 | 4.41 | 3.15 | SVR |
| 0.93 | 0.96 | 1.21 | 0.80 | 0.92 | 0.97 | 4.88 | 2.87 | RF |
| 0.95 | 0.99 | 1.36 | 0.01 | 0.90 | 0.99 | 4.54 | 1.58 | XGB |
| 0.96 | 0.99 | 0.92 | 0.20 | 0.93 | 0.99 | 4.28 | 0.78 | CB |
|  |  | **BY** |  |  | **SY** |  |  |  |
| 0.31 | 0.54 | 3.28 | 2.46 | 0.96 | 0.97 | 1.18 | 1.05 | Linear |
| 0.97 | 0.98 | 0.66 | 0.53 | 0.99 | 0.99 | 0.44 | 0.36 | Ridge |
| 0.97 | 0.98 | 0.66 | 0.53 | 0.99 | 0.99 | 0.45 | 0.35 | Lasso |
| 0.95 | 0.97 | 0.90 | 0.63 | 0.99 | 0.99 | 0.34 | 0.28 | EN |
| 0.23 | 0.53 | 3.40 | 2.39 | 0.99 | 0.99 | 0.43 | 0.33 | SVR |
| 0.31 | 0.70 | 3.29 | 1.96 | 0.98 | 0.99 | 0.66 | 0.51 | RF |
| 0.69 | 0.98 | 2.23 | 0.50 | 0.97 | 1.00 | 0.94 | 0.00003 | XGB |
| 0.86 | 0.99 | 1.46 | 0.17 | 0.98 | 0.99 | 0.64 | 0.12 | CB |
|  |  | **PY** |  |  |  | **PP** |  |  |
| 0.95 | 0.974 | 3.05 | 2.31 | 0.90 | 0.94 | 2.64 | 2.08 | Linear |
| 0.99 | 0.99 | 0.30 | 0.22 | 0.99 | 0.99 | 0.31 | 0.23 | Ridge |
| 0.99 | 0.99 | 0.30 | 0.24 | 0.99 | 0.99 | 0.30 | 0.22 | Lasso |
| 0.99 | 0.99 | 0.39 | 0.38 | 0.98 | 0.99 | 0.97 | 0.86 | EN |
| 0.99 | 0.99 | 0.39 | 0.2 | 0.96 | 0.97 | 1.64 | 1.31 | SVR |
| 0.98 | 0.99 | 1.79 | 1.36 | 0.96 | 0.98 | 1.66 | 1.11 | RF |
| 0.99 | 1.00 | 1.35 | 0.0003 | 0.96 | 0.99 | 1.53 | 0.40 | XGB |
| 0.98 | 0.99 | 1.66 | 0.24 | 0.96 | 0.99 | 1.65 | 0.23 | CB |
|  |  |  |  |  |  | **HI** |  |  |
|  |  |  |  | 0.88 | 0.89 | 1.24 | 1.01 | Linear |
|  |  |  |  | 0.99 | 0.99 | 0.34 | 0.24 | Ridge |
|  |  |  |  | 0.99 | 0.99 | 0.33 | 0.26 | Lasso |
|  |  |  |  | 0.98 | 0.99 | 0.40 | 0.29 | EN |
|  |  |  |  | 0.99 | 0.99 | 0.32 | 0.24 | SVR |
|  |  |  |  | 0.79 | 0.88 | 1.78 | 1.16 | RF |
|  |  |  |  | 0.97 | 1.00 | 0.60 | 0.002 | XGB |
|  |  |  |  | 0.93 | 0.99 | 0.94 | 0.20 | CB |
