# Supplementary material for: Machine learning-based evaluation of seed priming and biostimulant applications in rainfed wheat
Source: PeerJ. 2026 Mar 2;14:e20578. doi: 10.7717/peerj.20578 (PMC12962134; doi:10.7717/peerj.20578)
Supplement: Supplemental Information 2 [file peerj-14-20578-s002.docx]

| **Model** | **Hyperparameter** | **Values / Range** |
| --- | --- | --- |
| Ridge | alpha | 50 values from 10−410^{-4} to 10410^{4} (log scale) |
| Lasso | alpha | 50 values from 10−410^{-4} to 10410^{4} (log scale) |
| ElasticNet | alpha | [0.01, 0.1, 1.0] |
|  | l1_ratio | [0.1, 0.5, 0.9] |
| SVR | C | [1.0, 10.0, 100] |
|  | gamma | ['scale', 'auto'] |
|  | epsilon | [0.1, 0.2, 0.5] |
|  | kernel | ['linear', 'poly', 'rbf', 'sigmoid'] |
| Random Forest | n_estimators | [50, 60, 80, 100, 200, 300] |
|  | max_depth | [None, 1, 2, 3, 4, 5, 6, 7, 10, 15, 20] |
|  | min_samples_split | [2, 3, 4, 5, 6] |
|  | min_samples_leaf | [3, 5, 7, 9, 11] |
|  | max_features | ['sqrt', 'log2', None] |
| XGBoost | n_estimators | [100, 300, 500] |
|  | learning_rate | [0.01, 0.1, 0.3] |
|  | max_depth | [3, 4, 5, 6] |
|  | subsample | [0.5, 0.7, 1.0] |
|  | colsample_bytree | [0.5, 0.7, 1.0] |
|  | booster | ['gbtree', 'dart'] |
| CatBoost | iterations | [100, 200, 300] |
|  | depth | [4, 6, 10, 12, 15] |
|  | learning_rate | [0.01, 0.05, 0.1, 0.2] |
|  | l2_leaf_reg | [1, 3, 5, 7, 9] |
|  | bagging_temperature | [0, 0.5, 1] |
|  | border_count | [32, 50, 100] |
