# Supplementary material for: Machine learning-based evaluation of seed priming and biostimulant applications in rainfed wheat
Source: PeerJ. 2026 Mar 2;14:e20578. doi: 10.7717/peerj.20578 (PMC12962134; doi:10.7717/peerj.20578)
Supplement: Supplemental Information 3 [file peerj-14-20578-s003.docx]

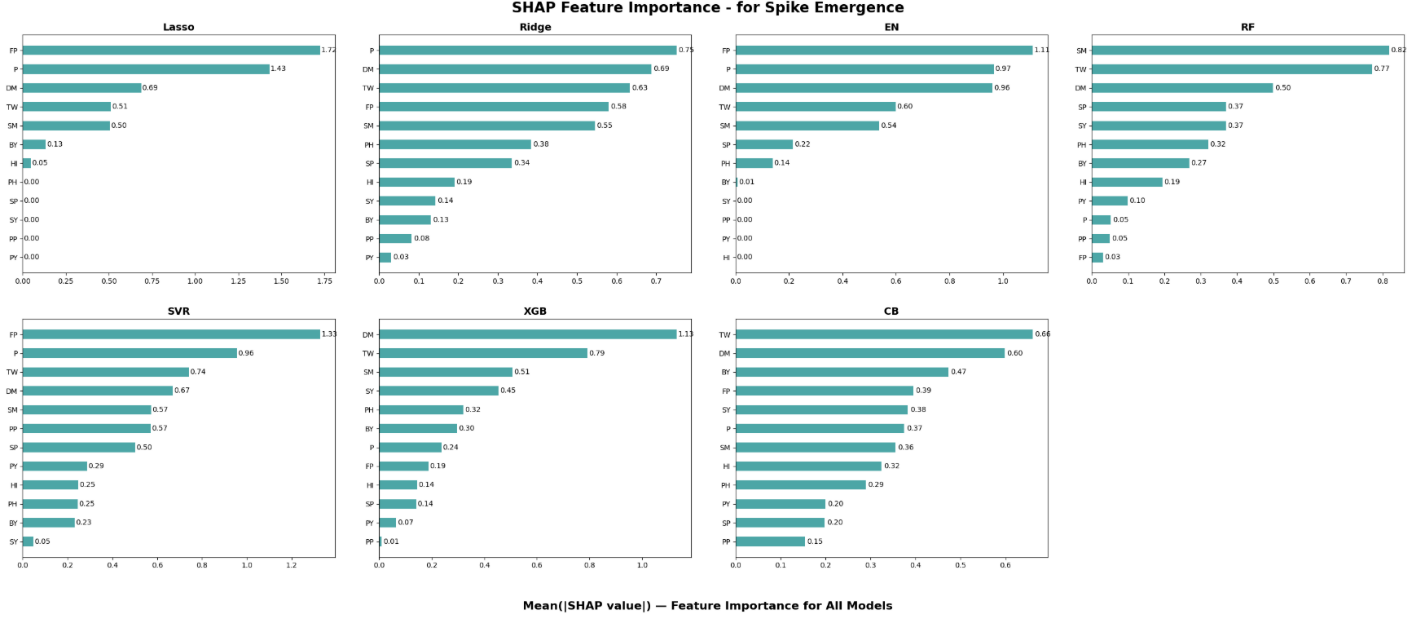
 Figure S1. SHAP Feature Importance for Spike Emergence


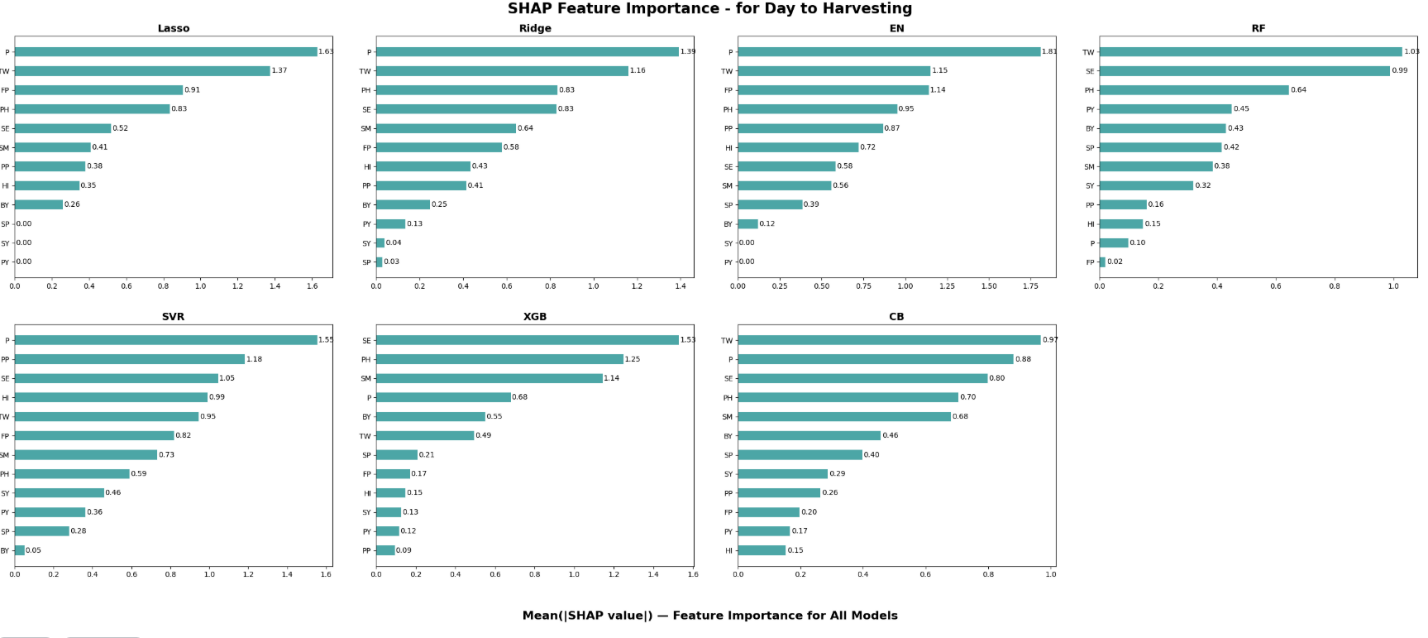


Figure S2. SHAP Feature Importance for Day To Harvesting


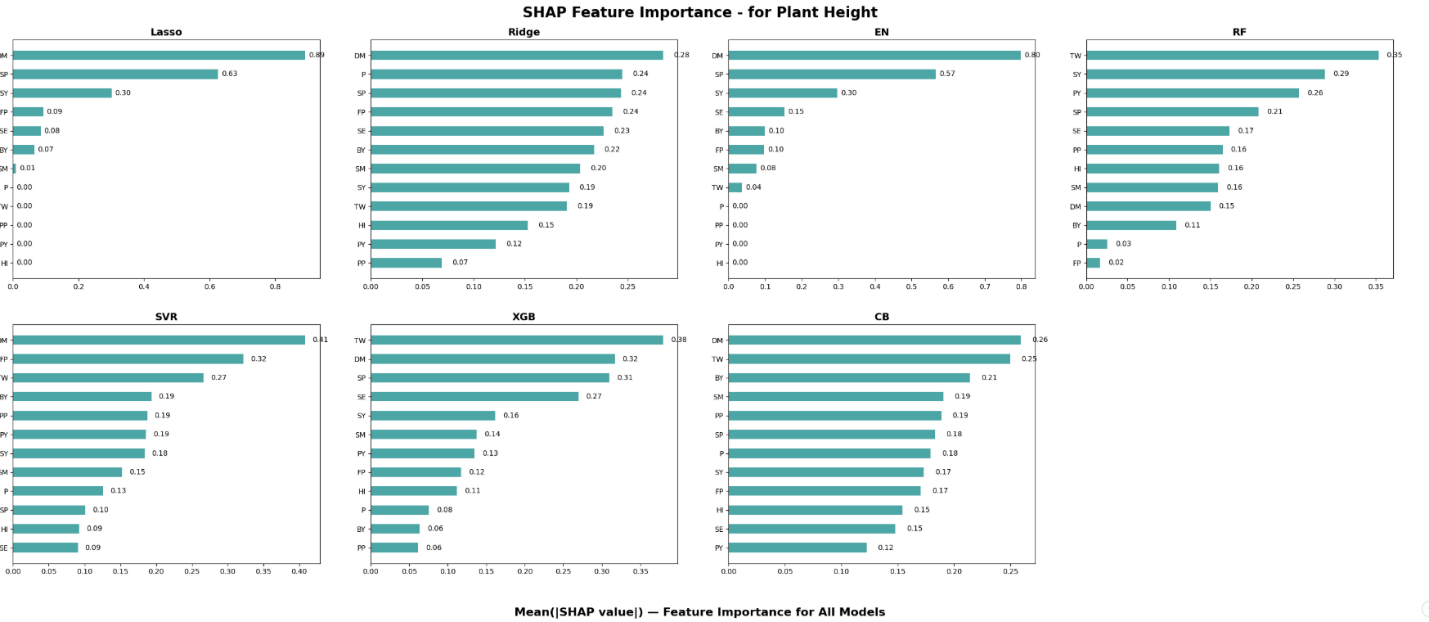


Figure S3. SHAP Feature Importance for Plant Height


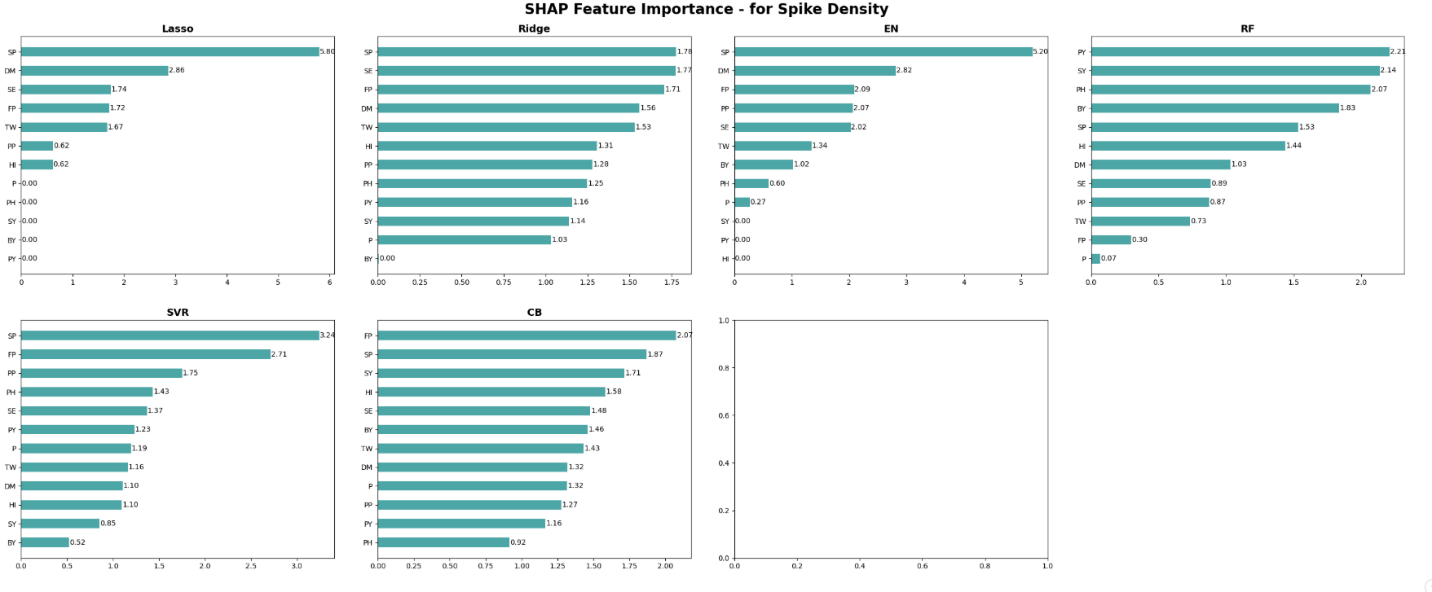
 Figure S4. SHAP Feature Importance for Spike Density


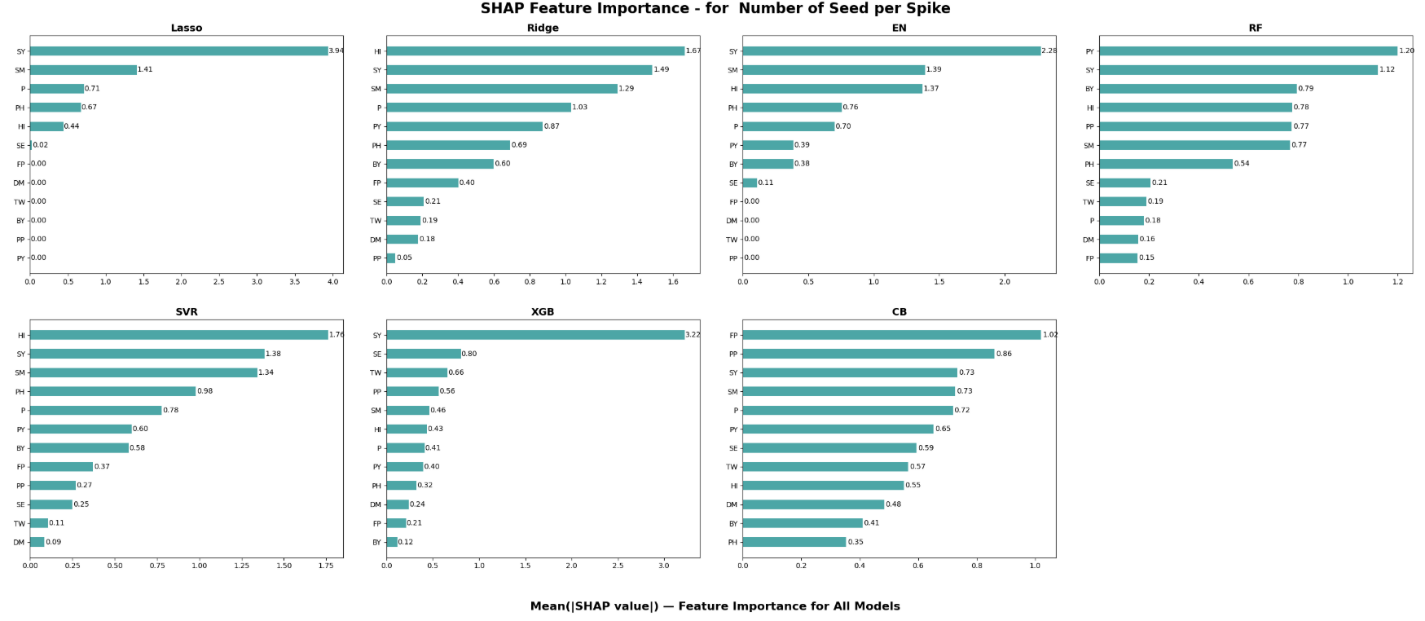


Figure S5. SHAP Feature Importance for Number of Seed per Spike


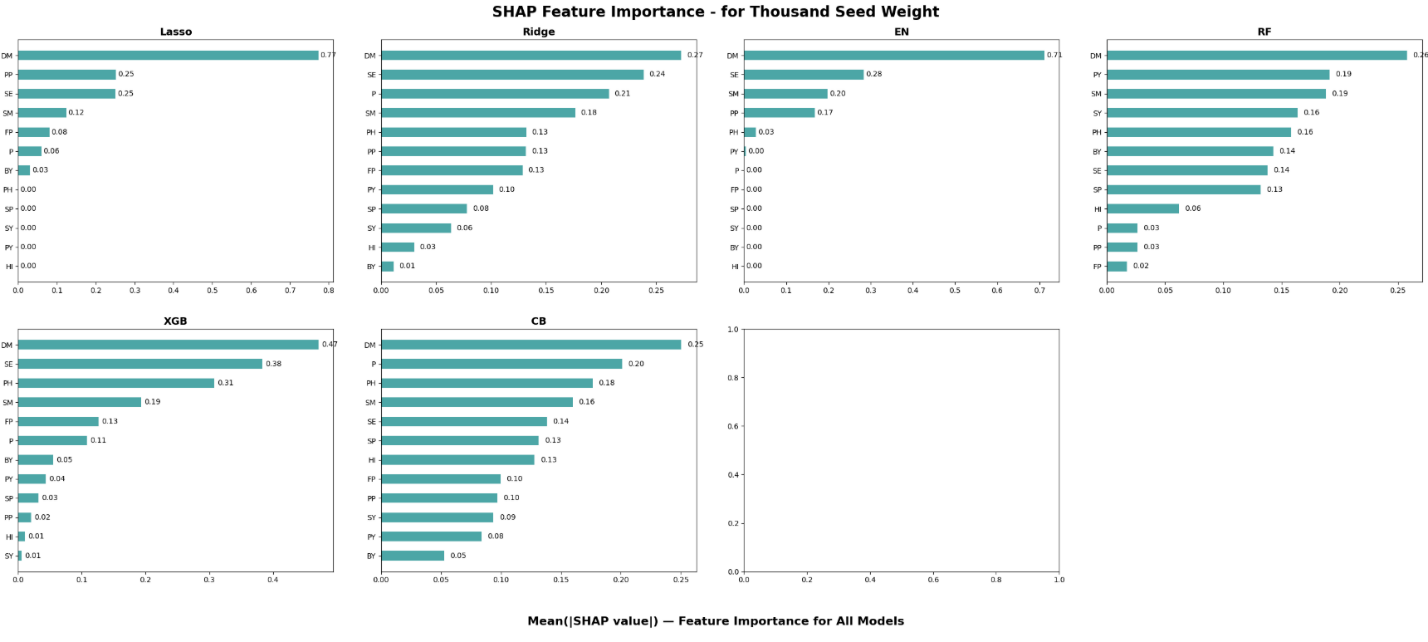
 Figure S6. SHAP Feature Importance for Thousand Seed Weight


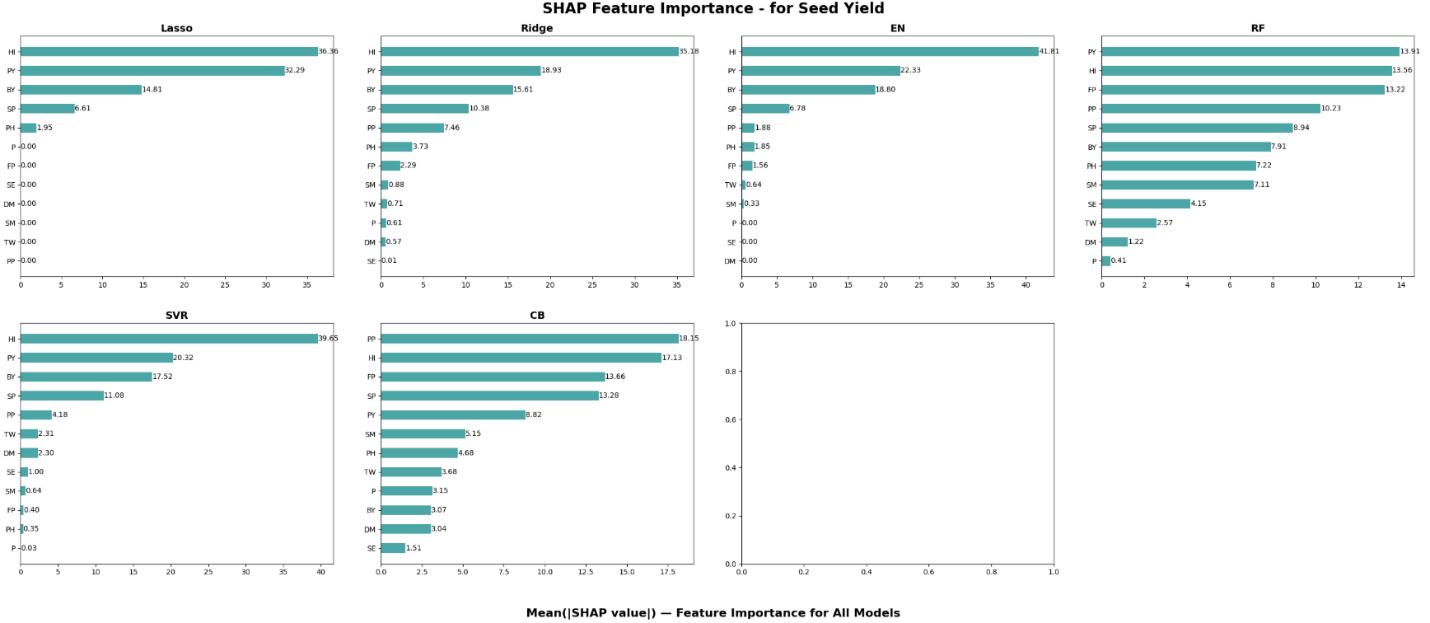


Figure S7. SHAP Feature Importance for Thousand Seed Yield


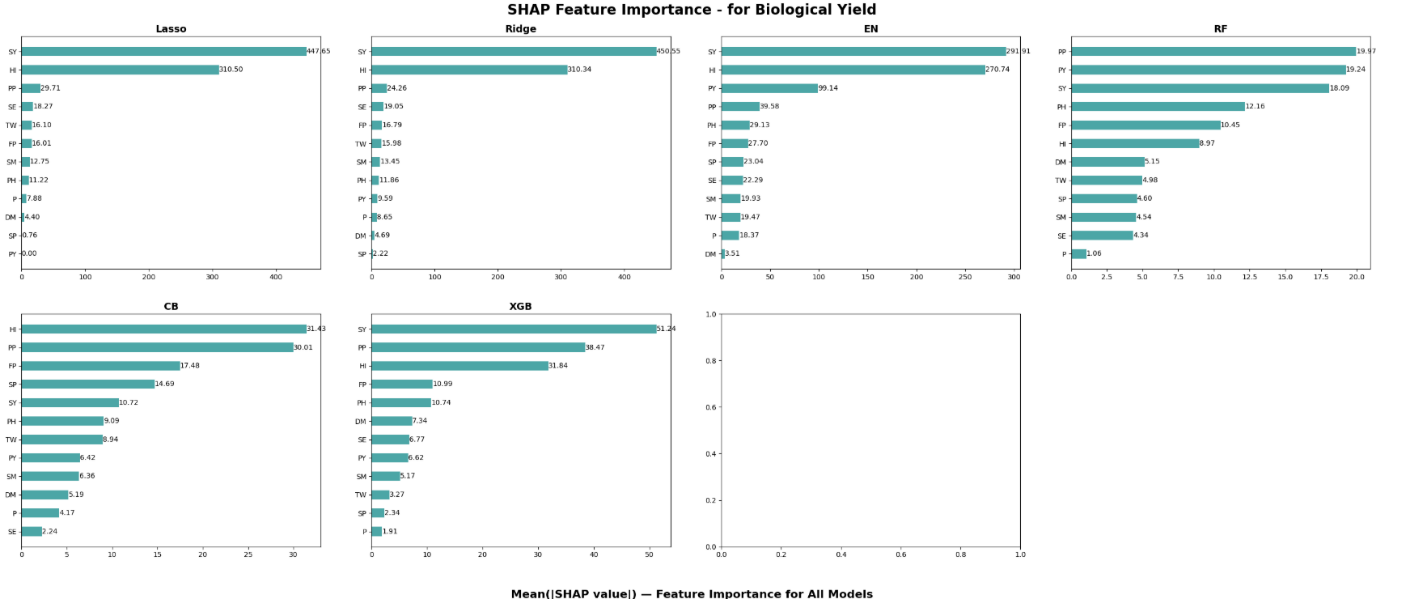


Figure S8. SHAP Feature Importance for Biological Yield


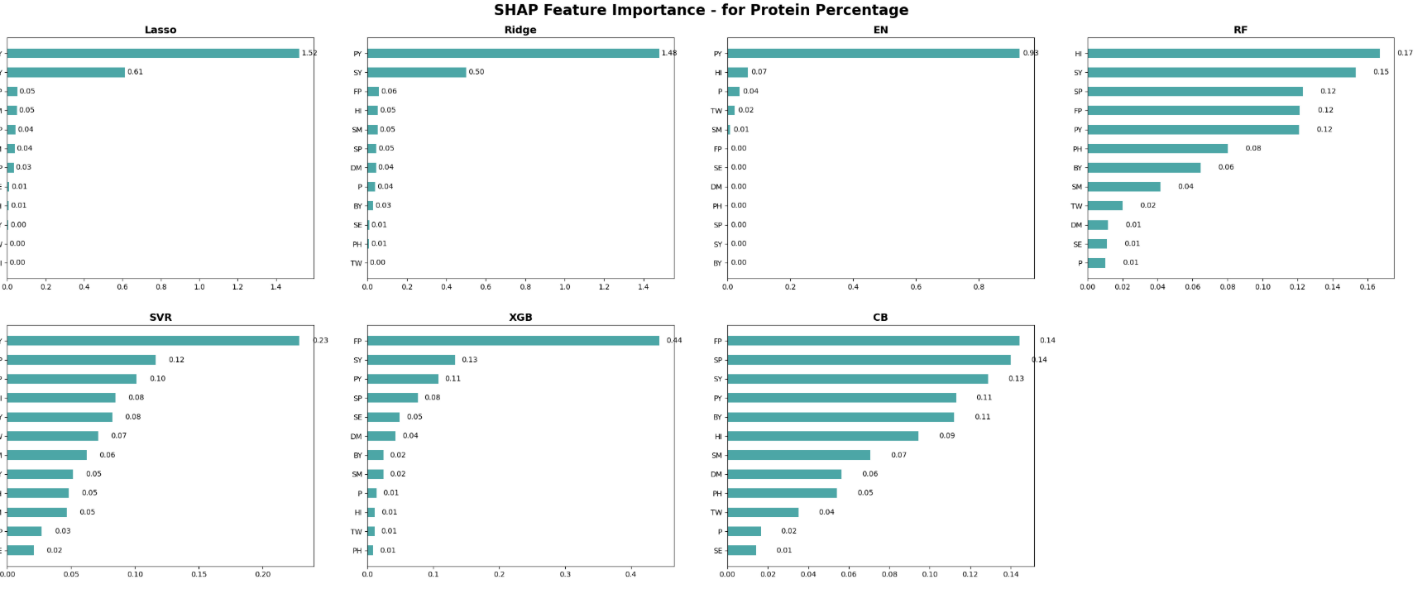


Figure S9. SHAP Feature Importance for Protein Percentage


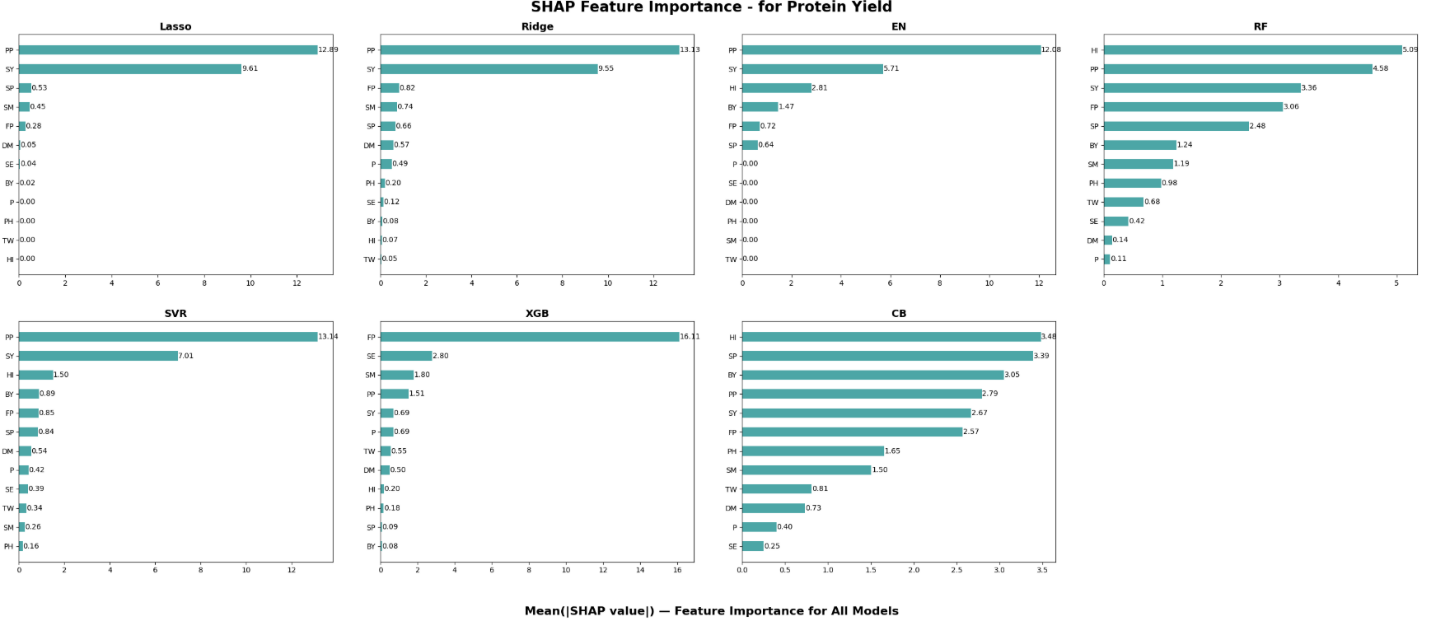


Figure S10. SHAP Feature Importance for Protein Yield


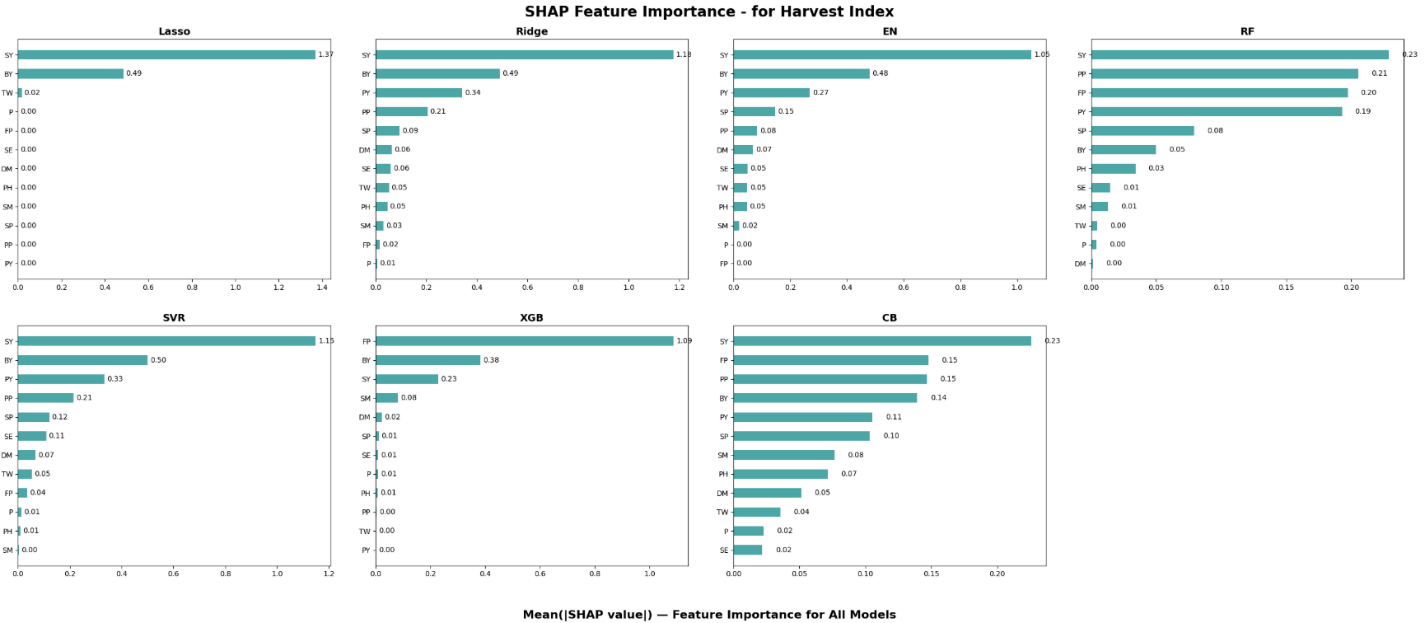


Figure S11. SHAP Feature Importance for Harvest Index
